# cGAS/STING signaling pathway in gynecological malignancies: From molecular mechanisms to therapeutic values

**DOI:** 10.3389/fimmu.2025.1525736

**Published:** 2025-01-30

**Authors:** Danyang Zhang, Bingxue Zhang

**Affiliations:** Department of Obstetrics, The First Hospital of China Medical University, Shenyang, Liaoning, China

**Keywords:** DNA damage response, cGAS, immunotherapy, cervical cancer, ovarian cancer, endometrial cancer, STING, PARPi

## Abstract

Gynecological cancers, including cervical, ovarian, and endometrial malignancies, remain a significant global health burden, exacerbated by disparities in access to preventive measures such as HPV vaccination and routine screening. The cGAS/STING signaling pathway, a pivotal mechanism in innate immunity, detects cytosolic DNA from pathogens or cellular damage, triggering immune responses via type I interferons and inflammatory cytokines. This pathway’s dual role in gynecological cancers, either promoting antitumor immunity or facilitating tumor immune evasion, makes it a compelling target for innovative therapies. The article outlines cGAS/STING’s influence on tumor microenvironments, immune surveillance, and inflammation, with emphasis on molecular mechanisms driving cancer progression. It explores interactions between DNA damage response pathways and immune modulation, highlighting the impact of cGAS/STING activation or suppression in ovarian, cervical, and endometrial cancers. The therapeutic potential of STING agonists, PARP inhibitors, and targeted immunotherapies is reviewed, demonstrating how these approaches can boost immune responses, counteract chemotherapy resistance, and improve patient outcomes. The study also discusses strategies for leveraging cGAS/STING signaling to enhance the efficacy of immunotherapies and address tumor-mediated immune suppression, providing insights into future directions for personalized cancer treatments.

## Introduction

In 2022, cervical, ovarian, and endometrial cancers remained the most prevalent gynecological cancers worldwide. Cervical cancer, strongly linked to HPV, had a higher impact in regions with lower scores on the Human Development Index (HDI), a measure assessing levels of health, education, and income, where limited access to HPV vaccines and routine screening contribute to elevated incidence and mortality rates (1, 2). Ovarian cancer, meanwhile, was more frequent in higher-HDI regions, as was endometrial cancer, which also had a notable presence in these areas. These differences reflect significant gaps in preventive services and early detection, emphasizing the need for global initiatives to reduce mortality, especially in areas with fewer resources (3). Identifying new signaling pathways and immune-targeted strategies in gynecological cancer therapy is crucial because these approaches address the limitations of existing treatments, such as resistance to chemotherapy and hormone therapy, as well as poor survival rates in advanced stages. By targeting specific molecular pathways involved in cancer progression, such as PI3K/AKT/mTOR, Notch, and Wnt, and leveraging immune responses with checkpoint inhibitors like PD-1/PD-L1 and CTLA-4, these therapies can halt tumor growth, reduce recurrence, and improve patient outcomes (4). The cGAS/STING pathway is a crucial immune response mechanism that identifies DNA in the cytoplasm, often from pathogens or cellular damage, and triggers immune defenses, including the release of type I interferons and other inflammatory signals (5). The enzyme cyclic GMP-AMP synthase (cGAS) detects foreign or abnormal DNA in the cytoplasm and produces a signaling molecule called cyclic GMP-AMP (cGAMP) (6). This molecule then binds to and activates the stimulator of interferon genes (STING) protein, initiating a cascade of immune responses (7). This pathway has recently attracted attention in cancer therapy due to its unique capacity to stimulate antitumor immunity by activating immune cells and promoting tumor cell death, while also enhancing the effects of traditional treatments like chemotherapy and radiotherapy (8, 9). However, cGAS/STING can sometimes aid tumor growth and metastasis, highlighting the importance of targeted therapeutic use. As a result, research has focused on developing STING agonists to boost immune attacks on cancer and STING inhibitors to reduce unwanted inflammation, positioning the pathway as a promising avenue in cancer treatment advancements (10). A recent review has highlighted that the cGAS-STING pathway is crucial in immune regulation within the female reproductive system, where its activation both aids immune defense and drives inflammatory responses associated with certain diseases. In endometriosis and adenomyosis, overactivation of this pathway leads to chronic inflammation, promoting abnormal cell growth and tissue invasion. In cancers like ovarian, cervical, and endometrial cancer, however, cGAS-STING is often suppressed, allowing tumor cells to evade immune detection (11). While the article outlines these functions in cancer, it provides limited detail, suggesting that a deeper review is needed to fully understand cGAS-STING’s roles and therapeutic potential in reproductive cancers. The aim of our study will be to elucidate the complex interplay between various DNA damage response mechanisms and immune signaling pathways, with a focus on the activation and regulation of cGAS-STING, NF-κB, and related pathways in gynecological cancers. Our study will further investigate the impact of targeted therapies, including STING agonists, PARP inhibitors, and natural compounds, on tumor immune dynamics and treatment efficacy, ultimately providing insights into strategies for enhancing immunotherapeutic responses and overcoming resistance in these cancers.

## Overview of cGAS-STING pathway

The cGAS-STING pathway is an essential immune response mechanism that detects cytosolic DNA, often from pathogens or damaged cells, triggering inflammation. When cGAS binds to foreign or misplaced DNA, it synthesizes the signaling molecule cGAMP, which then activates the STING protein on the endoplasmic reticulum membrane. This activation causes STING to relocate to the Golgi, where it recruits the kinase TBK1, which phosphorylates IRF3, a transcription factor. Phosphorylated IRF3 then enters the nucleus to stimulate the production of type I interferons and other inflammatory cytokines. These signaling molecules amplify the immune response, activating nearby cells and attracting immune cells to clear infections or cellular debris (12). The cGAS-STING pathway’s role in cancer is complex, functioning as both a tumor suppressor and promoter. On the one hand, it stimulates antitumor immunity by enhancing type I interferon production, cellular senescence, apoptosis, and autophagy, facilitating immune surveillance and tumor eradication. On the other hand, chronic activation can foster tumor progression by creating an immunosuppressive microenvironment, promoting epithelial-mesenchymal transition, and increasing genomic instability through impaired DNA repair mechanisms. This duality is influenced by factors such as tumor type, the spatial and temporal activation of cGAS-STING, and the surrounding tumor microenvironment (13). The “double-edged sword” effect of the cGAS-STING pathway could be attributed to its canonical and non-canonical activations. This pathway, initially known for its role in immune defense, has context-dependent outcomes in cancer (14). Canonical activation occurs when cGAS senses cytosolic dsDNA, catalyzing the production of cGAMP. This activates STING, leading to the induction of interferons and pro-inflammatory genes via IRF3 and NFκB pathways. These signals can suppress tumorigenesis by promoting immune surveillance and tumor clearance. However, chronic activation may paradoxically create an immunosuppressive tumor microenvironment, promoting metastasis and immune evasion (15)​. Non-canonical activation bypasses cGAS, involving alternative pathways such as ATM, PARP1, or IFI16, which activate STING independently. This can lead to NFκB-driven responses and other downstream effects that may either restrict or promote cancer progression, depending on the context. Genes involved in these pathways include those encoding cGAS, STING, TBK1, IRF3, and NFκB, as well as SASP-associated genes like IL-6 and CXCL8, which are linked to senescence and inflammation​ (14, 16).

The cGAS-STING pathway exhibits a complex relationship with metastasis, acting as both a driver and potential therapeutic target. Chronic activation of cGAS-STING in tumors with high chromosomal instability (CIN) promotes metastasis through noncanonical NF-κB signaling, leading to the secretion of pro-inflammatory cytokines, immune suppression, and evasion of oncogene-induced senescence. Additionally, tumor cells can exploit cGAMP transfer through gap junctions to adjacent stromal cells, such as astrocytes, which activate STING and secrete inflammatory cytokines, further facilitating metastatic spread. This interplay between tumor-intrinsic and nonautonomous mechanisms highlights the dual roles of cGAS-STING in both reinforcing tumor progression and shaping the tumor microenvironment to support metastatic growth. Understanding these dynamics may guide therapeutic interventions to selectively target its prometastatic activities while preserving its antitumor immune functions (14, 17, 18). MicroRNAs (miRNAs) play a pivotal role as upstream regulators of the cGAS-STING pathway, significantly impacting its function in both physiological and pathological states. By targeting mRNAs of core components like cGAS, STING, and downstream molecules such as TBK1 and IRF3, miRNAs modulate the pathway’s activation, influencing immune responses (19). For instance, miRNAs like miR-25 and miR-93 suppress cGAS expression, facilitating tumor progression (20), while others like miR-27b-3p dampen STING activity to mitigate inflammation (21). This regulatory capacity offers a promising avenue for therapeutic interventions. For cancer patients, strategies like using miRNA mimics or inhibitors could fine-tune cGAS-STING signaling to enhance anti-tumor immunity while minimizing pro-tumorigenic effects (19). In this section, we review each steps of the activation of cGAS-STING pathway:

### DNA sensing by cGAS

**Figure 1:**
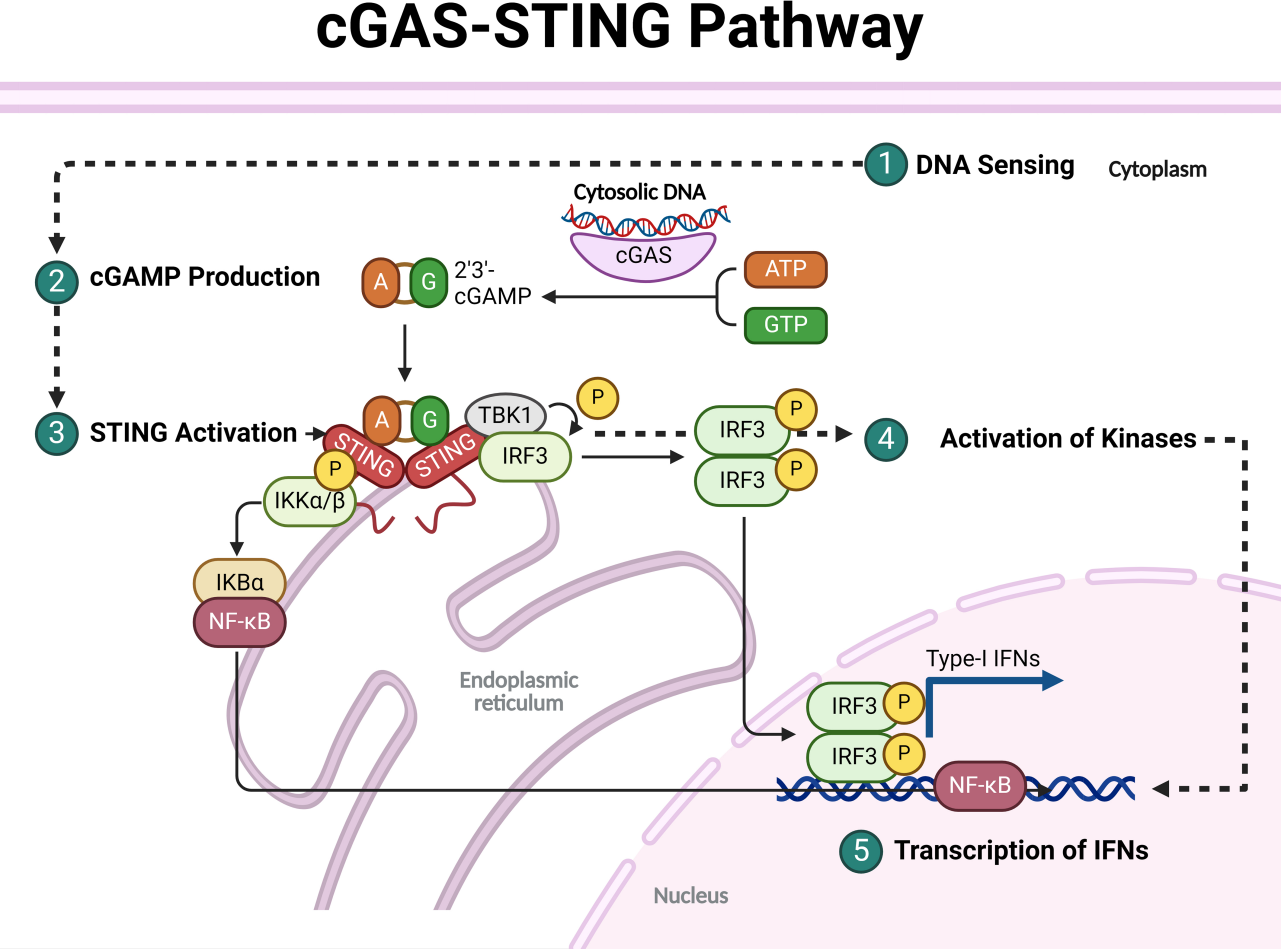
Overall mechanisms involved in activating the cGAS-STING pathway.

cGAS is a critical enzyme in the innate immune response, responsible for detecting cytosolic DNA, which may originate from pathogens (such as viral or bacterial DNA) or damaged host cells (self-DNA). Upon binding to cytosolic double-stranded DNA (dsDNA) in a length-dependent manner, cGAS undergoes conformational changes that activate its catalytic site, synthesizing the cyclic dinucleotide 2′3′-cGAMP from ATP and GTP (**Figure 1**) [Reviewed by (22)].

### Production of cGAMP

The production of cGAMP is essential as it acts as a critical second messenger in the immune response, activating the STING pathway. When cGAS (cyclic GMP–AMP synthase) detects cytosolic DNA, often from pathogens or damaged host cells, it synthesizes cGAMP, which then binds to and activates STING located on the endoplasmic reticulum. cGAMP is also unique because it can be transferred to adjacent cells through gap junctions or transporters, extending its immune-activating effects beyond the cell where it was produced. This ability makes cGAMP a pivotal immunotransmitter, impacting various immune functions, including antitumor responses and inflammatory regulation, thus playing a critical role in both innate immunity and in the pathogenesis of inflammatory and autoimmune diseases (**Figure 1**) [Reviewed by (23)].

### Activation of STING and recruitment and activation of kinases

Activation of STING is triggered when cGAMP, produced by the enzyme cGAS upon binding to dsDNA, binds to STING at the ER membrane. This binding causes STING to undergo significant conformational changes, leading to its oligomerization, release from the ER, and recruitment of TANK-binding kinase 1 (TBK1). STING then moves through the ER-Golgi intermediate compartment (ERGIC) to the Golgi apparatus, where TBK1 phosphorylates both STING and interferon regulatory factor 3 (IRF3). This phosphorylation enables IRF3 to dimerize and translocate to the nucleus, initiating the transcription of type I interferons and other immune-stimulating genes. Additionally, STING activation can drive NF-κB activation and trigger autophagy, all of which contribute to a robust immune response. In the cGAS-STING pathway, IκB kinase (IKK) plays a crucial role in the activation of proinflammatory signaling. I addition to TBK1, upon STING translocates from the endoplasmic reticulum to the Golgi, it recruits and activates IKK. IKK is particularly important for the activation of the nuclear factor kappa-light-chain-enhancer of activated B cells (NF-κB) pathway. Once activated, IKK phosphorylates the inhibitor of NF-κB (IκB), leading to its degradation and allowing NF-κB to translocate into the nucleus. This translocation results in the transcription of proinflammatory cytokines and other immune-related genes, contributing to the innate immune response. Additionally, IKK, together with TBK1, supports the activation of IRF3, amplifying the production of type I interferons essential for antiviral and antitumor immunity (**Figure 1**) [Reviewed by (24, 25)].

### Transcriptional activation of interferon and pro-inflammatory genes and immune response

Upon the transcription of type I interferons (IFN-Is) and other immune-stimulating genes, a robust antiviral and pro-inflammatory response is initiated. IFN-Is, primarily IFN-α and IFN-β, are secreted and bind to the interferon alpha receptor (IFNAR) on both the producing and neighboring cells, triggering a cascade that activates the JAK-STAT signaling pathway. This activation leads to the expression of interferon-stimulated genes (ISGs), which encode proteins that inhibit viral replication, enhance antigen presentation, and bolster the activity of immune cells such as natural killer (NK) cells and macrophages. In parallel, other cytokines and chemokines are produced, which recruit and activate additional immune cells to the site of infection, creating a highly coordinated immune defense network. This response, while crucial for controlling infections, must be tightly regulated to prevent excessive inflammation, which can result in tissue damage and contribute to autoimmune disease if dysregulated (**Figure 1**) [Reviewed by (26)].

## Molecular mechanisms of cGAS-STING in gynecological cancers

The cGAS and STING pathway is a critical cellular mechanism for detecting cytosolic DNA, leading to the activation of immune responses that can have both tumor-suppressive and tumor-promoting effects. By exploring the roles of cGAS-STING in ovarian, endometrial, and cervical cancers, we will discuss how this pathway contributes to immune surveillance, inflammation, and the tumor microenvironment, offering insights into potential therapeutic targets for improving cancer treatment outcomes (18).

### cGAS triggering in gynecological cancers

**Figure 2:**
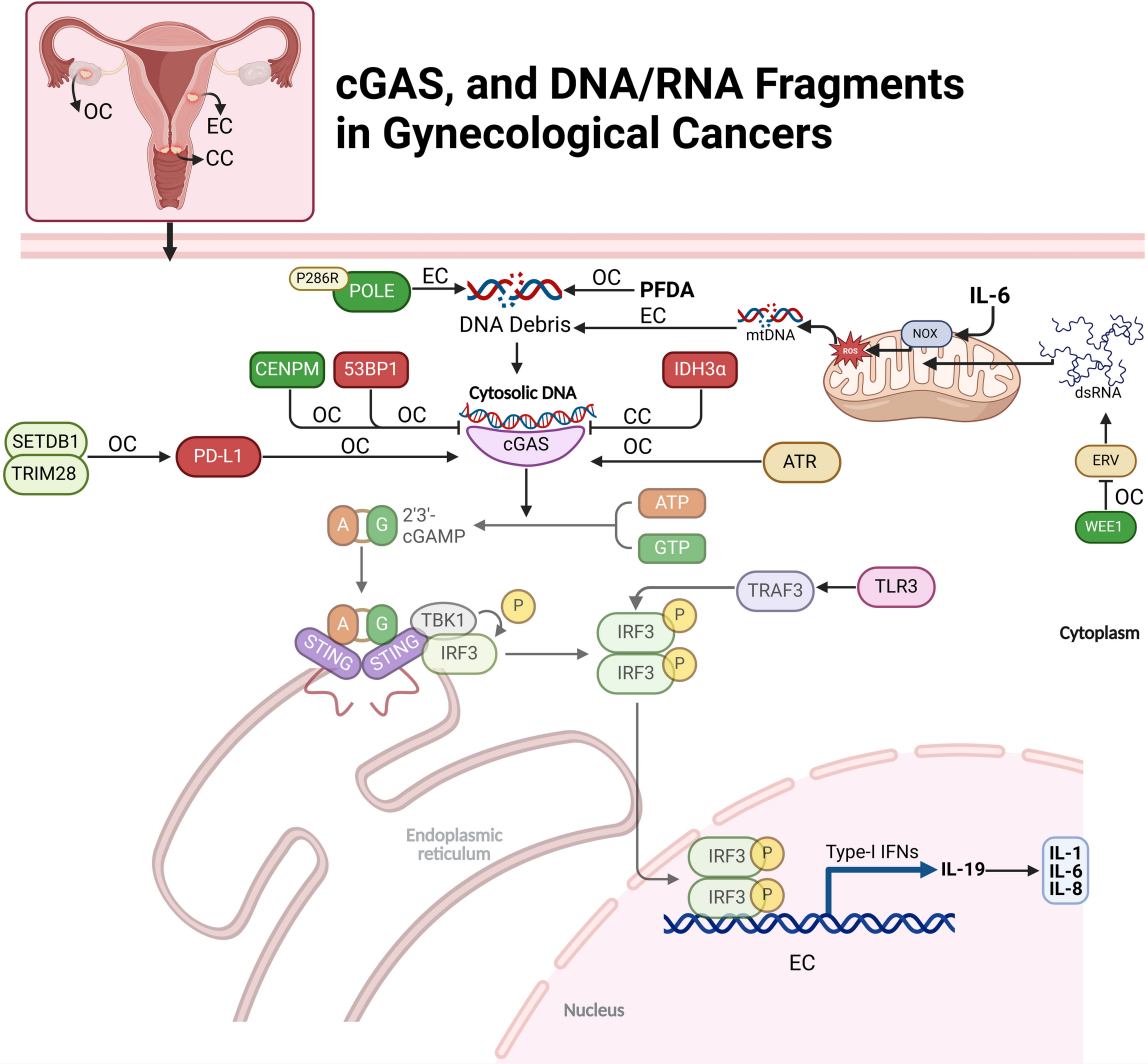
Regulation of cGAS and DNA/RNA fragments in gynecological cancers. This figure depicts the cGAS-STING pathway’s role in ovarian (OC), endometrial (EC), and cervical cancers (CC). The pathway is initiated by the recognition of cytosolic DNA, leading to immune responses through various downstream signals and modulators. Key molecular factors, including DNA debris, mtDNA, and dsRNA, activate cGAS-STING, triggering immune responses like cytokine production (e.g., IL-6, IL-1). Cancer-specific interactions, such as IDH3α in CC and POLE mutations in EC, influence the pathway’s activation and immune modulation.

Methylation in the cGAS gene promoter region has been linked to the risk of cervical precancerous lesions (CPL) and cervical cancer (CC) in a Southern Chinese population. cGAS hypermethylation is significantly higher in CPL patients than in healthy controls, suggesting it may be an early event in cervical disease progression. Women with cGAS promoter methylation have a 2.49 times higher risk of CPL. A synergistic interaction between high-risk HPV infection and cGAS methylation further increases CPL risk. However, no significant association is found between cGAS methylation and CC, indicating it may be a marker for early CPL detection rather than advanced cervical cancer (27). In cervical cancer, IDH3α suppresses the cGAS–STING pathway, impairing the immune response within the tumor microenvironment. High IDH3α expression creates an acidic environment that limits immune cell infiltration and activity, promoting tumor growth. Inhibition of IDH3α activates the cGAS–STING pathway, increasing immune-stimulating cytokines and enhancing CD8+ T cell infiltration. This activation boosts anti-tumor immunity and sensitizes cancer cells to chemoimmunotherapy, making IDH3α a potential therapeutic target for improving cervical cancer treatment efficacy (28). The loss of 53BP1 in ovarian cancer cells enhances cGAS-dependent antitumor immunity by enabling excessive DNA end-resection during double-strand break repair, generating cytoplasmic DNA fragments. These fragments activate the cGAS-STING pathway, triggering pro-inflammatory cytokine release and recruiting immune cells, including CD8+ T cells, macrophages, and natural killer cells, to the tumor microenvironment. This immune infiltration boosts the anti-tumor response and increases the tumor’s sensitivity to immune checkpoint blockade therapy, improving the likelihood of a successful immune response (29). Reducing CENPM expression in ovarian cancer (OC) cells activates the cGAS-STING pathway, suppressing tumor growth and spread by inducing pyroptosis, an inflammatory cell death. CENPM, a highly expressed hub gene in OC, shows promise as a diagnostic marker. Silencing CENPM significantly impaired OC cell proliferation, migration, and invasion while increasing pyroptosis-related proteins and inflammatory cytokines. The anti-tumor effects of CENPM knockdown were reversed by cGAS inhibition, confirming cGAS-STING involvement (30). The SETDB1–TRIM28 complex suppresses antitumor immunity in ovarian cancer by downregulating PD-L1 and inhibiting CD8+ T cell infiltration in the tumor microenvironment. This complex silences immune-stimulating genes, reducing tumor visibility to immune cells. Inhibiting SETDB1–TRIM28 disrupts this suppression, increasing PD-L1 expression and activating the cGAS–STING pathway via micronuclei formation. This activation induces a type I interferon response, enhancing immune cell infiltration and activity. In a mouse model, SETDB1 knockout boosted PD-L1 levels and CD8+ T cell infiltration, increasing tumor sensitivity to anti-PD-L1 immune checkpoint therapy (**Figure 2**) (31).

### DNA debris in gynecological cancers

In ovarian cancer cells, IL-19 expression is activated by the JNK and cGAS-STING pathways in response to DNA damage, driving cytokine production. DNA damage induces IL-19 through ROS-mediated JNK signaling and cytoplasmic DNA detection via the cGAS-STING pathway, independent of IL-1R or p38 MAPK signaling. IL-19 enhances the production of IL-1, IL-6, and IL-8, as its inhibition significantly reduces their levels. Pathway activation varies by DNA damage type: ionizing radiation (IR) relies on ROS and JNK, while ATR kinase inhibition predominantly activates cGAS-STING. Notably, IL-19 amplifies cytokine responses without affecting cGAS-STING-mediated PDL1 expression, underscoring its specific role in cytokine regulation (32). The P286R mutation in DNA polymerase ϵ (POLE) activates immune pathways in endometrial cancer (EC) by impairing DNA repair and causing genomic instability. This mutation reduces POLE’s proofreading ability, accumulating DNA damage and releasing cytoplasmic DNA fragments. These fragments activate the cGAS-STING pathway, inducing inflammatory responses via TBK1 phosphorylation and IRF3 activation. Consequently, POLE mutations elevate pro-inflammatory cytokines and chemokines, enhancing cytotoxic T cell recruitment and activation. *In vitro* and *in vivo* models show that POLE mutations suppress tumor growth, reduce stemness, and increase T-cell infiltration, promoting an intrinsic immune response against tumor progression (33). Perfluorodecanoic acid (PFDA), a persistent synthetic PFAS chemical, induces DNA damage in ovarian epithelial cells by causing double-strand breaks (DSBs) and disrupting DNA repair. At low doses, PFDA promotes nuclear accumulation of cGAS, which binds to DNA damage sites and interferes with homologous recombination (HR), a high-fidelity DNA repair process, by blocking key repair proteins from accessing the damaged DNA (**Figure 2**) (34).

### mtDNA in gynecological cancers

Interleukin-6 (IL6) facilitates immune escape in endometrial carcinoma (EC) by inducing mtDNA leakage and activating the cGAS-STING pathway. IL6 upregulates NADPH oxidase (NOX), increasing ROS levels and causing oxidative stress that damages mitochondrial membranes. This leads to mtDNA release into the cytoplasm, where it activates cGAS. The cGAS-STING pathway triggers TBK1 and IRF3 phosphorylation, stimulating interferon (IFN) production and upregulating PD-L1, a key immunosuppressive molecule (35). IL6-treated EC cells release extracellular vesicles (EVs) containing elevated levels of PD-L1 and mtDNA. These EVs induce apoptosis in cytotoxic CD8+ T cells while protecting regulatory CD4+/CD25+ T cells, weakening the anti-tumor immune response and aiding tumor immune escape. IL6-induced mtDNA leakage and activation of the cGAS-STING pathway help EC cells evade immune detection, promoting tumor progression. This highlights the potential of targeting IL6 or the cGAS-STING axis as therapeutic strategies in EC (**Figure 2**) (36).

### dsRNA in gynecological cancers

dsRNA) influences the cGAS-STING pathway indirectly through RIG-I-like receptors (RLRs), rather than directly activating cGAS. While cGAS typically responds to cytoplasmic DNA to trigger STING and interferon responses, RNA viruses producing dsRNA can induce mitochondrial DNA release into the cytoplasm, which cGAS recognizes as a DAMP. This leads to indirect activation of the cGAS-STING pathway in response to RNA virus infections. To evade immune detection, RNA viruses often degrade or inhibit cGAS and STING components (37). The study by Guo et al. investigates how inhibiting the WEE1 kinase, which regulates cell cycle checkpoints and DNA repair, can activate anti-tumor immunity in ovarian cancer. WEE1 inhibition increases endogenous retroviral elements (ERVs), triggering a dsRNA stress response and activating immune pathways, including IFN signaling, to enhance T cell activity even in tumors lacking the cGAS-STING pathway. The research found that WEE1 inhibition elevates IFN-responsive genes and PD-L1 expression, improving the effectiveness of immune checkpoint blockade (ICB) targeting PD-L1. Combining WEE1 inhibitors with ICB reduced tumor growth in a CD8+ T cell-dependent manner. The study suggests that measuring FOXM1, SETDB1, and IFN-stimulated genes could help identify patients likely to benefit from this combination therapy, supporting further clinical trials (**Figure 2**) (38). Theodoraki et al. have examined that how different forms of dsRNA influence immune responses in the ovarian cancer tumor microenvironment (TME) by selectively activating distinct pathways. Both poly-I:C and rintatolimod activate TLR3-mediated nuclear translocation of TRAF3 and IRF3, leading to type-1 interferon production and CXCL10 expression, which attract CD8+ T cells for anti-tumor immunity. However, only poly-I:C activates the helicase/MAVS pathway, triggering NFκB and inducing immunosuppressive factors like COX2 and chemokines that attract Tregs, promoting tumor growth. Rintatolimod bypasses this pathway, enhancing immune activation without suppressive effects. This selective activation suggests targeting TLR3/TRAF3/IRF3 without activating NFκB could improve cancer immunotherapy by boosting anti-tumor immunity while minimizing immune suppression (39).

### STING in gynecological cancers

In gynecological cancers, STING expression is notably high, particularly in cervical adenocarcinoma (90%) and serous high-grade ovarian cancer (86%), suggesting a strong presence of this immune sensor. Elevated STING levels correlate with adverse clinical phenotypes, indicating a link to aggressive tumor behavior. However, despite high STING expression, there is no significant association with CD8+ lymphocyte density, implying limited type I interferon production. STING expression is strongly linked to PD-L1 expression, suggesting an immune-regulatory role. This association points to the potential of combining STING pathway agonists with PD-L1 inhibitors to enhance immune responses in gynecological cancers with high STING expression [Described by (40)].

#### STING in ovarian cancer

**Figure 3:**
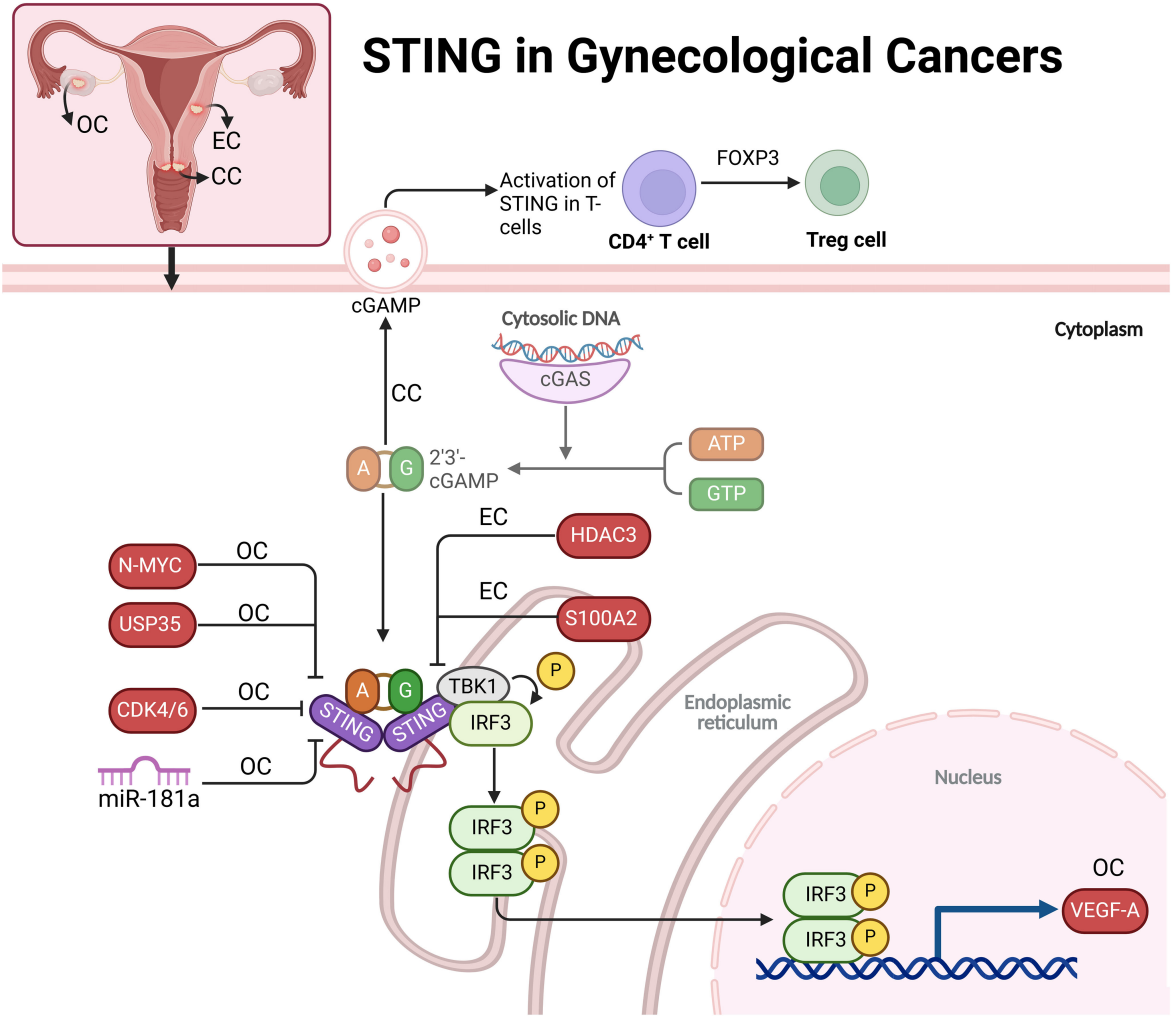
The role of STING in gynecological cancers, including ovarian (OC), cervical (CC), and endometrial (EC) cancers. In these cancers, cytosolic DNA activates the cGAS-STING pathway, leading to immune responses that are modified by various regulators like N-MYC, USP35, CDK4/6, HDAC3, and S100A2. STING activation promotes immune signaling but also contributes to immune evasion mechanisms, such as angiogenesis via VEGF-A in ovarian cancer and Treg cell expansion in cervical cancer. This highlights STING’s dual role in promoting both immune response and tumor progression, suggesting it as a potential therapeutic target in gynecological cancers.

BRCA1-deficient ovarian cancers exhibit chromatin instability, leading to the release of dsDNA into the cytoplasm, which activates the STING pathway. This pathway triggers inflammation, upregulating type I interferons and attracting T cells to the tumor microenvironment, establishing baseline immunoreactivity. However, STING-mediated inflammation also increases VEGF-A expression, promoting angiogenesis and immune escape. Thus, while STING activation initially aids immune cell recruitment, it paradoxically enhances immune resistance, allowing the tumor to evade immune attack. This dual role of STING presents both challenges and opportunities for therapeutic strategies targeting BRCA1-deficient ovarian cancers (41). CDK4/6 is overexpressed in OC tissues, contributing to an immunosuppressive tumor environment and poor prognosis by suppressing immune cell infiltration and antigen presentation. The CDK4/6 inhibitor palbociclib activates immunity by enhancing IFN secretion and immune-stimulating gene expression via the STING pathway. This activation boosts antigen presentation, promoting an anti-tumor immune response. These findings suggest that CDK4/6 inhibitors, especially through the STING pathway, could enhance immune responses in OC, providing a dual approach to tumor growth suppression and immune activation (42). Deubiquitinase USP35 inhibits STING-mediated interferon signaling in ovarian cancer by binding to and deubiquitinating STING, preventing its activation. STING typically triggers immune responses by producing type I interferons that attract immune cells like CD8+ T cells. However, in ovarian cancer, USP35 is upregulated, leading to STING inactivation and immune suppression. When STING is phosphorylated, it binds to USP35, which removes activating ubiquitin tags, impairing STING’s ability to activate the TBK1-IRF3 signaling pathway. This reduces interferon production and immune cell infiltration, allowing cancer cells to evade the immune system (43). In high-grade serous ovarian carcinoma (HGSC), the N-MYC oncogene disrupts innate immune signaling by inhibiting key pathways involved in immune response activation, particularly type I IFN signaling. N-MYC suppresses the cGAS-STING and RIG-I-like receptor (RLR) pathways, which detect cytoplasmic DNA and RNA to initiate immune responses. In the STING pathway, N-MYC impairs STING oligomerization, preventing its translocation to the Golgi apparatus, where it activates TBK1 and induces IFN production. N-MYC also disrupts the RIG-I/MAVS pathway by blocking MAVS aggregation and mitochondrial localization, reducing IFN production. This dual inhibition leads to decreased type I IFNs and ISGs, limiting immune cell recruitment and promoting an immune-evasive, “cold” tumor phenotype. In patient tumor samples, a high N-MYC signature correlates with reduced IFN pathway activation. N-MYC’s repression of innate immune signaling contributes to immune evasion in HGSC and may serve as a target for immunotherapy (44). In BRCA-mutated ovarian cancer, miR-181a promotes resistance to poly (ADP-ribose) polymerase inhibitors (PARPi) by targeting the STING pathway. High levels of miR-181a in PARPi-resistant cells reduce STING expression, impairing downstream signaling necessary for cytokine production and IFNG responses. This dampens the immune response, allowing cancer cells to survive despite PARPi treatment. Additionally, miR-181a can be transferred through extracellular vesicles, spreading resistance within the tumor microenvironment by suppressing STING in neighboring cells (**Figure 3**) (45).

#### STING in cervical cancer

Researchers have studied the impact of STING1 gene variants, specifically homozygous HAQ/HAQ and R232H/R232H, on cervical cancer outcomes. While these variants were not more common in cervical cancer patients than in controls, homozygous variants were associated with worse disease-specific survival (DSS), higher recurrence rates, and earlier diagnoses, particularly in adenocarcinoma cases. These findings suggest impaired immune responses due to a dysfunctional cGAS-STING pathway, leading to more aggressive disease despite early detection. Additionally, the V48V variant, although functionally neutral, may serve as a surrogate marker for poor prognosis, identifying high-risk patients (46). Activating STING can suppress cervical cancer tumor growth by enhancing the body’s anti-tumor immune response. In cervical cancer, STING signaling is often reduced, impairing immune detection and response to tumor cells. Using the STING agonist ADU-S100, researchers showed that STING activation decreases cancer cell viability, increases immune-enhancing cytokines IFNβ and IL-6, and activates the TBK1/NF-κB pathway, boosting immune activity. In animal models, ADU-S100 treatment reduced tumor size by promoting the infiltration of CD8+ T cells and CD103+ dendritic cells, crucial for effective immune defense. These findings suggest STING activation as a promising immunotherapeutic approach for cervical cancer (47). In cervical cancer, particularly HPV-positive cases, T cell-intrinsic STING signaling promotes immunosuppression by enhancing the differentiation of CD4+ T cells into regulatory T cells (Tregs) through FOXP3 upregulation. Tumor-derived exosomes (T-EXOs), containing immune-modulatory molecules like TGF-β and cGAMP, activate STING in tumor-infiltrating lymphocytes by delivering tumor-associated DNA and proteins. This activation, independent of traditional type I interferon pathways, triggers phosphorylation of transcription factors SMAD3 and STAT5, which are essential for FOXP3 transcription. FOXP3 drives Treg expansion, reducing cytotoxic CD8+ T cells and fostering a more immunosuppressive tumor microenvironment. This mechanism highlights T cell-intrinsic STING as a potential target for cancer immunotherapy (**Figure 3**) (48). High STING levels correlate with improved survival rates in patients treated with surgery or radio(chemo)therapy, indicating STING’s role as an independent prognostic marker. CD103, marking tumor-reactive T cells, shows additional prognostic value, especially in patients with advanced disease undergoing radiotherapy, where high CD103+ T cell infiltration combined with elevated STING levels associates with better disease-free and disease-specific survival. Together, STING and CD103 may offer a powerful approach for risk stratification in cervical cancer, going beyond conventional clinical metrics (49).

#### STING in endometrial cancer

S100A2 acts as an oncogene in endometrial cancer, promoting tumor aggressiveness through enhanced cell migration, invasion, and EMT. It is upregulated in cancer tissues and linked to poorer prognosis. Knockdown of S100A2 activates the cGAS/STING pathway, suppressing tumor cell viability and invasiveness, suggesting that S100A2 typically inhibits this pathway. Inhibiting the STING pathway reverses the effects of S100A2 knockdown, highlighting the pathway’s role in tumor progression. These findings suggest S100A2 as a potential therapeutic target, where its inhibition could reduce tumor growth and improve patient outcomes by harnessing the STING pathway’s anti-tumor immunity (50). In endometrial cancer, Histone Deacetylase 3 (HDAC3) plays a pivotal role in promoting tumor growth by repressing STING expression through epigenetic mechanisms. HDAC3, likely influenced by β-estradiol-ERα signaling, deacetylates histone H3 at the STING promoter, suppressing STING transcription. This repression allows for increased cell proliferation and decreased apoptosis. Restoring STING expression by inhibiting HDAC3 with RGFP-966 leads to reduced tumor growth and enhanced cell apoptosis. These findings suggest that HDAC3 inhibitors, possibly in combination with STING activators, could be a promising therapeutic strategy to improve immune response and inhibit tumor progression in endometrial cancer (**Figure 3**) (51).

### TBK1 in gynecological cancers

#### TBK1 in cervical cancer

**Figure 4:**
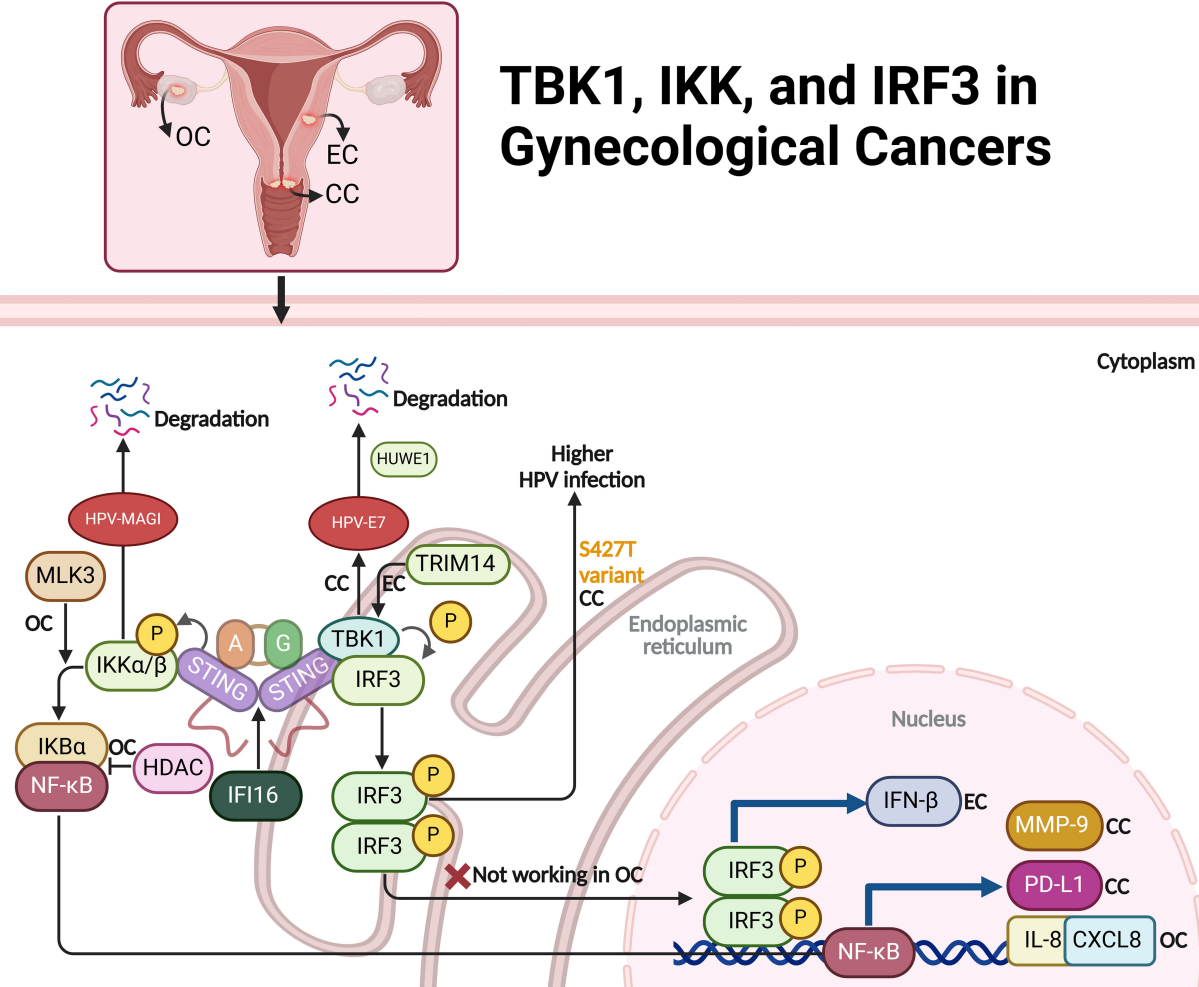
The role of TBK1, IKK, and IRF3 signaling pathways in ovarian (OC), endometrial (EC), and cervical cancer (CC). In cervical cancer, STING/TBK1 activation triggers the degradation of HPV E7 oncoproteins, reducing tumor growth and potentially enhancing radiotherapy efficacy. IFI16 further promotes PD-L1 expression, facilitating immune evasion via the STING-TBK1-NF-κB pathway. In endometrial cancer, TCF19 and TRIM14-mediated TBK1-IRF3 activation increases IFN-β production, leading to immune exhaustion. In ovarian cancer, MLK3 modulates IKK activity, influencing NF-κB signaling, while IKK-ϵ enhances metastasis, and HDAC inhibition upregulates IL-8/CXCL8 through an IKK-dependent mechanism. IRF3 pathway dysregulation in ovarian cancer impairs immune responses, contributing to immune evasion. The figure also notes a genetic variant (S427T) in IRF3 linked to persistent HPV infection in cervical cancer, underlining the importance of these pathways in immune response and cancer progression.

Activating the STING/TBK1 pathway in cervical cancer cells induces the degradation of HPV16 and HPV18 E7 oncoproteins, which are critical for the tumorigenic properties of HPV-related cancers. Through the phosphorylation of E7 proteins at specific serine residues, STING/TBK1 activation promotes their ubiquitination and subsequent degradation by the E3 ligase HUWE1. This degradation not only inhibits tumor cell proliferation and survival but also enhances the effectiveness of radiotherapy by reducing the required doses for similar therapeutic outcomes. This approach opens promising new treatment strategies for HPV-positive cervical cancer, directly targeting viral oncoproteins and potentially enhancing therapies like radiotherapy or therapeutic vaccines (52). IFI16 plays a significant role in promoting cervical cancer progression by upregulating PD-L1 expression in the tumor microenvironment through the activation of the STING-TBK1-NF-κB signaling pathway. In HPV-positive cervical cancer, IFI16 levels are abnormally elevated, leading to increased PD-L1 expression in cancer cells. This occurs when IFI16 activates the STING-TBK1 pathway, which in turn activates NF-κB. NF-κB binds to the PD-L1 promoter, driving its upregulation. The resulting increase in PD-L1 expression enables cancer cells to evade immune detection by engaging immune checkpoints that inhibit T cell function. Experimental knockdown of IFI16 reduces PD-L1 levels and suppresses cancer cell proliferation, migration, and invasion in both *in vitro* and *in vivo* models. By promoting tumor progression and contributing to an immunosuppressive microenvironment, IFI16 presents a potential therapeutic target to restore immune surveillance and improve treatment outcomes in HPV-positive cervical cancer (**Figure 4**) (53).

#### TBK1 in endometrial cancer

Ma et al. have explored how the transcription factor TCF19 contributes to immune exhaustion in microsatellite-instable (MSI)-EC through the activation of the TRIM14-mediated TBK1-IRF3 and NF-κB pathways, which enhance the production of IFN-β. High TCF19 expression leads to increased transcription of TRIM14, which in turn activates the TBK1 pathway, a key component of the cGAS-STING signaling axis. This pathway responds to cytosolic DNA accumulation, which occurs due to DNA repair defects in MSI cancers. The continuous activation of the TBK1-IRF3 pathway results in elevated IFN-β levels, which promote CD8+ T cell exhaustion within the tumor microenvironment. As a result, the immune response is dampened, facilitating tumor progression (**Figure 4**) (54).

### IKK in gynecological cancers

#### IKK in ovarian cancer

IKK-ϵ is a key regulator of tumor invasion and metastasis in ovarian cancer. Its elevated expression is mainly seen in metastatic tumors, suggesting its role in disease progression. IKK-ϵ regulates genes involved in motility and inflammation, promoting cell invasion and dissemination. Depleting IKK-ϵ in ovarian cancer cells reduces growth, adhesion, and invasiveness, while overexpression in less aggressive cells increases metastatic potential (55). MLK3 regulates the NF-κB pathway in ovarian cancer by inhibiting IKK activity. In SKOV3 cells, MLK3 prevents IKK from phosphorylating and degrading IκBα, inhibiting NF-κB activation. Silencing MLK3 increases IKK activity, leading to IκBα degradation and NF-κB activation, promoting cell survival and resistance to apoptosis. Overexpressing MLK3, whether wild-type or kinase-dead, reduces IKK activity, stabilizing IκBα and inhibiting NF-κB activation, making cells more sensitive to apoptosis induced by etoposide (56). HDAC inhibition in ovarian cancer cells activates a signaling pathway that increases IL-8/CXCL8 expression through an IKK-dependent mechanism. Agents like vorinostat cause histone acetylation and p65 NFκB accumulation in the nucleus, promoting a relaxed chromatin structure at the IL-8 promoter. This allows IKKα recruitment and phosphorylation of NFκB components, enhancing p65 NFκB binding and transcriptional activity at the IL-8 promoter. Acetylation of p65 NFκB further stabilizes its interaction with IL-8, driving its robust transcription. IL-8 upregulation promotes cancer cell survival and proliferation, counteracting the antitumor effects of HDAC inhibitors. This IKK-dependent IL-8 induction suggests combining HDAC inhibitors with IKK inhibitors may improve anticancer outcomes (**Figure 4**) (57).

#### IKK in cervical cancer

IKKβ plays a key role in cervical cancer by enhancing the oncogenic effects of HPV oncoproteins, especially E6, which degrades cellular proteins and disrupts cell integrity. As a regulator of NF-κB, IKKβ promotes inflammation, cell proliferation, and anti-apoptotic responses. In HPV-positive cells, IKKβ facilitates the degradation of PDZ domain proteins like Magi, contributing to cellular abnormalities. IKKβ also affects pathways involving the tumor suppressor p53. Inhibiting IKKβ suppresses HPV-induced transformation, blocking tumor growth and restoring cell polarity, making it a potential therapeutic target for HPV-related cervical cancer (58). Low doses of bisphenol A (BPA) promote cervical cancer cell migration and invasion by activating the IKKβ/NF-κB pathway. BPA exposure leads to IKKβ phosphorylation and NF-κB activation through p65 phosphorylation and nuclear translocation. Activated NF-κB increases the expression of migration-related proteins MMP-9 and fibronectin (FN), enhancing cancer cell motility. Inhibiting NF-κB with BAY 11-7082 or blocking IKKβ with specific inhibitors reduces BPA-induced upregulation of MMP-9 and FN, decreasing cell migration. This indicates that BPA induces cervical cancer cell migration via the IKKβ/NF-κB axis (59). Likewise, Kin17 plays a significant regulatory role in cervical cancer by influencing the activity of IKK (IκB kinase), which is crucial for cancer cell migration and invasion. In cervical cancer cells, knockdown of Kin17 results in reduced phosphorylation of IKKα, which in turn inhibits activation of the NF-κB pathway. This pathway is known to drive processes such as EMT, which is critical for cancer metastasis (60). By suppressing IKK phosphorylation, Kin17 knockdown reduces NF-κB activity and downregulates Snail, an EMT-associated transcription factor (**Figure 4**) (61).

### IRF3 in gynecological cancers

#### IRF3 in ovarian cancer

The STING/IRF3 pathway, crucial for innate immune responses to foreign DNA, is dysregulated in ovarian cancer. In normal ovarian epithelial cells, STING activates IRF3 upon recognizing cytosolic dsDNA, leading to IRF3 phosphorylation and subsequent transcription of type I interferons (IFN-α and IFN-β) and immune-related genes like PLSCR1. This enhances antiviral defense and activates pro-inflammatory pathways. However, in ovarian cancer cells, dsDNA transfection fails to trigger IRF3 phosphorylation or PLSCR1 induction, suggesting immune evasion by suppressing the STING/IRF3 pathway. This dysregulation likely contributes to immune evasion, allowing cancer cell proliferation without triggering antiviral and anti-tumor responses, highlighting the potential of STING/IRF3 modulation for restoring immune surveillance in ovarian cancer (**Figure 4**) (62).

#### IRF3 in cervical cancer

Variants in the FANCA gene, particularly the G501S polymorphism, are associated with an increased risk of cervical intraepithelial neoplasia grade 3 (CIN3) or cervical cancer. The IRF3 gene (S427T variant) is linked to a higher likelihood of persistent HPV infection, indicating its role in the immune response to HPV. These findings suggest that genetic variations in DNA repair and immune response pathways may contribute to HPV persistence and cervical cancer risk, warranting further investigation to confirm these associations and explore their mechanisms (**Figure 4**) (63).

## ISGylation in ovarian cancer

Madaan et al. investigated the role of ISGylation, where the ubiquitin-like protein ISG15 is attached to other proteins, in enhancing IFN and NFκB signaling in fallopian tube epithelial (FTE) cells, particularly with BRCA1 mutations. Using CRISPR to disrupt ISG15 and UBA7, they found ISGylation amplifies responses to dsRNA, activating IRF3 and NFκB via RIGI and MDA5 pathways, but not cGAS-STING. In BRCA1 mutation carriers, this could promote a proinflammatory environment, potentially contributing to the early stages of high-grade serous tubo-ovarian cancer (HGSTOC). Loss of ISGylation decreased cellular migration and stem cell gene activation, suggesting that elevated ISGylation supports malignancy. Targeting ISGylation, particularly inhibiting UBA7, may serve as a preventive strategy for HGSTOC in high-risk individuals by limiting proinflammatory signaling while preserving the beneficial roles of free ISG15 in immune response (64).

## cGAS-STING in HPV-induced cervical carcinogenesis

HPV is crucial in cervical cancer carcinogenesis, as its high-risk strains integrate into host DNA, causing persistent infection, oncoprotein expression, and disruption of cell cycle regulation (65). In HPV-associated cervical cancer, STING is expressed in basal cells where HPV infects and initiates carcinogenesis, and this expression is maintained through premalignant stages and into cancerous lesions. Activating STING with ligands triggers local inflammation and immune responses targeting HPV-infected and dysplastic cells, potentially leading to regression of premalignant lesions and helping control malignancies (66). In the Chinese population, susceptibility loci within the cGAS-STING pathway and the MHC gene interact with HPV infection to influence the risk of cervical precancerous lesions. The rs311678 polymorphism in the cGAS gene is protective, with individuals carrying the GG genotype having a significantly lower risk, likely due to enhanced immune response activation against HPV. Additionally, interactions between HPV infection, the cGAS variant, and age at menarche further modify this risk (67). Oncoproteins E6 and E7, expressed by high-risk HPV, transform normal cells into cancerous ones, especially in CC. They promote tumor progression by degrading tumor suppressors like p53 and disrupting cell cycle regulation. E6 and E7 also upregulate topoisomerase I (TOP1), an enzyme involved in DNA repair. This upregulation activates the cGAS-PD-L1 pathway, helping tumor cells evade immune detection. Specifically, TOP1 induces cGAS, which senses DNA breaks and activates downstream signaling, including NF-κB, leading to increased PD-L1 expression. PD-L1, a protein that binds to PD-1 on immune cells (68), effectively suppresses immune responses against the tumor, allowing cancer cells to grow unchecked (69). Activating the STING/TBK1 pathway suppresses tumor growth in HPV-positive cervical cancer by degrading HPV16/18 E7 oncoproteins. STING activation leads to TBK1 phosphorylation of Ser71 on HPV16 E7 and Ser78 on HPV18 E7, marking them for ubiquitination and degradation via HUWE1, reducing E7 levels and impairing tumor growth. This effect is independent of STING’s immune functions, indicating a direct anti-cancer mechanism. Combining STING activation with radiotherapy enhances this effect, suggesting STING agonists could be potent therapies, especially with radiation, for HPV-driven cervical cancer (52). High-risk HPV (hrHPV) E6 proteins inactivate IRF3 by binding to its LxxLL motifs and blocking phosphorylation sites needed for activation. Normally, IRF3 triggers interferon production in response to viral infection, but E6 binding masks key serine residues, preventing activation and suppressing the immune response. This helps the virus evade host defenses. Variations in E6 across hrHPV types can affect binding affinity and contribute to drug resistance, complicating treatment of different HPV strains (70). Lamin B1 (LMNB1) limits early HPV infection in cervical cancer cells by maintaining nuclear integrity and supporting autophagy. Reduced Lamin B1 causes nuclear envelope rupture, allowing more HPV particles to enter the nucleus. It also triggers persistent activation of cGAS, a DNA sensor that detects DNA leakage from frequent envelope disruptions, impairing autophagy and increasing viral accumulation. Thus, Lamin B1 restricts HPV infection by stabilizing the nuclear envelope and aiding autophagy, reducing viral access and persistence (71).

## cGAS-STING in therapy efficacy of gynecological cancers

**Figure 5:**
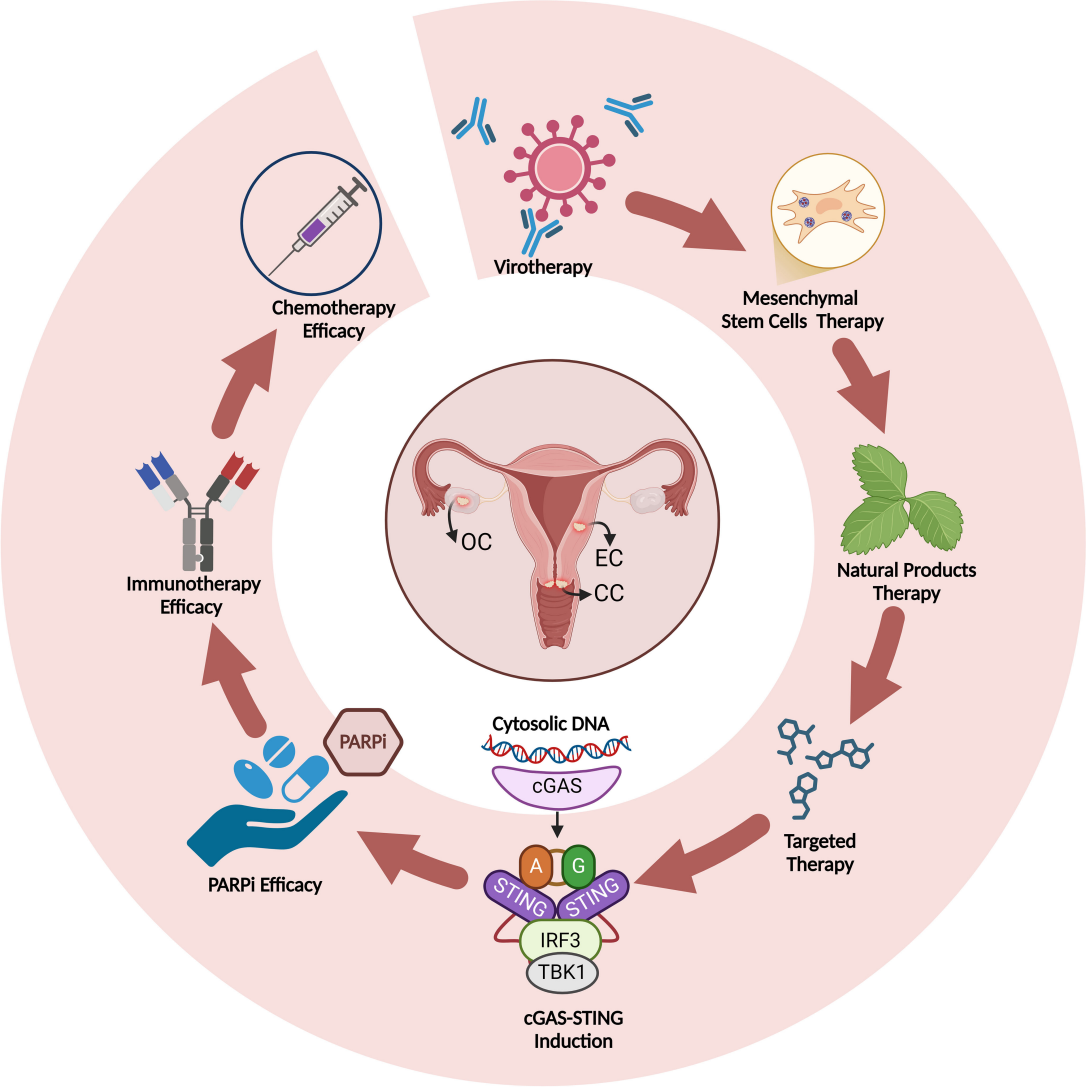
Induction of cGAS-STING by different therapeutics and improvement of therapy efficacy upon cGAS-STING induction in gynecological cancers.

The cGAS-STING pathway, usually involved in immune defense, contributes to cancer drug resistance by aiding cancer cell survival during chemotherapy and targeted treatments. DNA damage from drugs triggers cGAS-STING signaling, activating survival pathways that promote drug resistance. This suggests cancer cells exploit this immune response to protect against drug stress, and targeting the STING pathway could help overcome drug resistance in various cancers (**Figure 5**) (8, 72).

### cGAS-STING in chemotherapy efficacy of gynecological cancers

#### cGAS-STING in chemotherapy efficacy of ovarian cancer

Cisplatin demonstrates significant immune-modulatory effects in ovarian cancer mouse models by enhancing the immunogenicity of tumor cells and altering the tumor microenvironment, particularly through the upregulation of immune markers and pathways associated with inflammation (73). Grabosch and colleagues found that cisplatin-treated tumors show increased expression of calreticulin and MHC class I, enhancing immune cell recognition, particularly by T cells. They also discovered that cisplatin activates the cGAS/STING pathway, promoting pro-inflammatory signals and upregulating immunogenic markers. Chronic cisplatin exposure, especially in resistant tumor models, further amplifies cGAS/STING activation, increasing PD-L1, MHC class I, and calreticulin levels, making tumor cells more recognizable to immune cells (74). Cancer-associated fibroblasts (CAFs) promote platinum resistance in ovarian cancer by activating the cGAS-STING pathway after DNA transfer from cisplatin-exposed cancer cells. This activation increases IFNB1 production, enhancing DNA repair and reducing apoptosis, reinforcing chemotherapy resistance. High STING expression in CAFs is linked to poorer patient outcomes, especially in platinum-resistant cases. Inhibiting STING with H-151 increases ovarian cancer cells’ sensitivity to cisplatin, promoting DNA damage and suppressing tumor growth. Conversely, the STING agonist MSA-2 did not improve chemotherapy sensitivity, suggesting that STING inhibition, rather than activation, may be a more effective strategy to overcome platinum resistance (75). Exogenous activation of the STING pathway enhances chemotherapy response, especially in PTEN-deficient tumors, by transforming the immunosuppressive tumor immune microenvironment (TIME) into an immunostimulatory state. In PTEN-deficient HGSC, the TIME has high levels of M2-like macrophages and reduced CD8+ T-cell activation, contributing to chemoresistance. STING activation, combined with carboplatin chemotherapy, reprograms M2-like macrophages to an M1-like, tumor-fighting phenotype and restores CD8+ T-cell function, improving immune responses. This therapy increases cytotoxic immune cell recruitment and enhances survival, overcoming immune suppression in PTEN-deficient tumors. Thus, STING agonists could potentiate chemotherapy, making them promising for resistant tumor genotypes (76). Knockdown of TBK1 enhances paclitaxel sensitivity in ovarian cancer by stabilizing microtubules and disrupting cell cycle progression. TBK1 knockdown reduces MAP4 phosphorylation via inhibition of the p38 pathway, stabilizing microtubules and making them more responsive to paclitaxel, which induces apoptosis through microtubule stabilization. When combined with knockdown of another kinase like EDN2, TBK1 silencing further amplifies paclitaxel sensitivity, increasing apoptosis and cell-cycle arrest, thereby improving paclitaxel effectiveness in ovarian cancer treatment (77). Combining IKK inhibition with bortezomib (BZ) enhances ovarian cancer treatment by preventing the increase in IL-8 expression induced by BZ alone. IL-8, a pro-inflammatory chemokine, promotes tumor growth, survival, and metastasis. While BZ induces apoptosis, it paradoxically activates IL-8 via an IKK-dependent pathway, reducing its effectiveness. The IKK inhibitor Bay 117085 prevents this IL-8 increase, lowering IL-8 levels in tumor tissues and plasma, enhancing BZ’s pro-apoptotic effects, and significantly reducing tumor growth. This combination suppresses tumor-promoting inflammation, offering a promising therapeutic strategy (78).

### cGAS-STING in immunotherapy efficacy of gynecological cancers

#### cGAS-STING in immunotherapy efficacy of ovarian cancer

In BRCA1-deficient ovarian cancers, STING has a dual role in modulating the immune environment. STING activation triggers a type I interferon response, attracting CD8+ T cells into the tumor microenvironment, making the tumor more immunogenic. However, STING also upregulates VEGF-A, promoting angiogenesis and immune resistance. VEGF-A supports tumor growth by enhancing vascularization and inhibiting immune cell function, allowing the tumor to evade detection. This paradoxical role suggests that targeting both STING-induced VEGF-A production and boosting immune cell infiltration could improve therapies for BRCA1-mutant ovarian cancers (41). 53BP1 regulates DNA damage response (DDR) by preventing excessive DNA end resection, which limits tumor immunogenicity in BRCA1-deficient ovarian cancer. Loss of 53BP1 leads to “leaky” DNA end resection, causing the accumulation of cytoplasmic double-stranded DNA and micronuclei that activate the cGAS-STING pathway. This activation promotes immune cell infiltration, particularly CD8+ T-cells, into the tumor, increasing its visibility to the immune system. Ovarian tumors with low 53BP1 expression show improved responsiveness to immune checkpoint blockade (ICB), making 53BP1 loss a potential biomarker for predicting ICB therapy response (29). Protein Phosphatase 4 (PP4) regulates DNA damage response (DDR), cell cycle progression, and apoptosis, aiding cancer cells in DNA repair and chemotherapy resistance. Targeting PP4 with inhibitors like fostriecin or gene knockdown disrupts DNA repair, increasing DNA damage, especially when combined with carboplatin. This triggers inflammatory signaling, activating STAT1 and NF-κB, and promoting the production of pro-inflammatory cytokines (e.g., CCL5, CXCL10, IL-6), which recruit immune cells like CD8+ T cells and NK cells. PP4 inhibition also activates the STING pathway, boosting immune cell infiltration and NK cell-mediated cytotoxicity, reducing tumor growth (79).

#### cGAS-STING in immunotherapy efficacy of cervical cancer

IDH3α overexpression in cancers like uterine cervical cancer (UCC) and lung adenocarcinoma (LUAD) contributes to chemoimmunotherapy resistance by creating an acidic tumor microenvironment (TME) that impairs immune cell function, especially CD8+ T cells, and inhibits the cGAS–STING pathway. High IDH3α levels promote glycolysis and lactate production, leading to an acidic TME that supports cancer cell survival and suppresses immune responses. This environment, along with cGAS–STING inhibition, prevents immune cell infiltration and cytokine release necessary for an effective anti-tumor response. Targeting IDH3α can improve chemoimmunotherapy outcomes by reversing immunosuppressive conditions, enabling better CD8+ T cell infiltration, TME modulation, and immune signaling reactivation, making IDH3α a potential therapeutic target and biomarker for treatment response in IDH3α-overexpressing cancers (28).

### PARP inhibition response

#### PARP inhibition response in ovarian cancer

In ovarian cancer, STING counteracts PARP inhibitor (PARPi) resistance by reshaping the tumor microenvironment (TME). Activated STING switches immunosuppressive TAMs from a protumor (M2-like) to an antitumor (M1-like) state, enhancing PARPi efficacy. This activation boosts dendritic cell function, antigen presentation, and T-cell infiltration, strengthening antitumor immunity. STING agonism overcomes the STAT3-driven immunosuppressive TME in PARPi-resistant tumors, offering a promising strategy to restore PARPi sensitivity and improve outcomes in ovarian cancer (80). PARP inhibition in BRCA1-deficient ovarian cancer exploits synthetic lethality by targeting tumor cells’ reliance on PARP-mediated DNA repair and activates an immune response via the STING pathway. Olaparib induces DNA damage in BRCA1-deficient cells, releasing DNA fragments or cGAMP that are detected by antigen-presenting cells (APCs) like dendritic cells (DCs). This triggers the STING pathway, producing type I interferons and pro-inflammatory cytokines that activate CD4+ and CD8+ T cells. While olaparib increases PD-L1 expression on tumor cells, limiting immune activity, combining it with PD-1 blockade restores immune activation, improving antitumor efficacy. This dual approach makes PARP inhibitors and immune checkpoint inhibitors a promising combination for treating BRCA1-deficient ovarian cancer (81). Targeting GRB2 in combination with PARP inhibitors shows potential for enhancing immune response and reducing tumor burden, particularly in BRCA2-deficient ovarian cancers. GRB2 stabilizes RAD51 at stalled replication forks, preventing degradation by MRE11, thus maintaining genomic stability. GRB2 depletion in PARP-inhibited cells leads to DNA fragments accumulating in the cytoplasm, activating the cGAS–STING pathway and triggering an immune response. This combination promotes immune recognition, increases inflammatory cytokine release, and recruits cytotoxic T-cells to target cancer cells, reducing tumor growth and extending survival. This approach suggests GRB2 as a valuable target to sensitize BRCA2-deficient tumors to PARP inhibitors and improve immunotherapeutic outcomes (82). MiR-181a contributes to PARP inhibitor (PARPi) resistance in BRCA-mutated ovarian cancer by targeting and downregulating STING, a key protein in DNA damage response and immune activation. In BRCA-mutated cells, STING activation is crucial for inducing inflammatory cytokines and interferon responses that enhance PARPi effects. Upregulated miR-181a suppresses STING, reducing immune signaling and allowing cancer cells to evade PARPi’s cytotoxic effects. MiR-181a can also spread resistance through extracellular vesicles, transferring it to nearby non-resistant cells. This miR-181a-STING axis drives both PARPi resistance and cross-resistance to platinum-based chemotherapy, making it a promising biomarker and therapeutic target to overcome drug resistance in BRCA-mutated ovarian cancer (45).

## Targeting cGAS-STING in gynecological cancers

Targeting the cGAS-STING pathway in cancer therapy is promising due to its critical role in activating immune responses, particularly through type I interferon production, which stimulates T-cell priming and immune cell infiltration into tumors. By activating innate immunity, cGAS-STING signaling helps the immune system recognize and attack cancer cells, inhibiting tumor growth and improving patient outcomes. Therapies targeting this pathway include STING agonists, which enhance immune activation, and combination therapies with ICIs to convert “cold” tumors into “hot” ones. Additionally, STING-based vaccines and CAR-T cell therapies combined with STING agonists can boost immune recognition and tumor destruction, especially in tumors evading immune surveillance. These strategies offer significant potential for overcoming immune tolerance and improving cancer treatment efficacy (83, 84). In this section, we explore the effects of different treatment strategies in targeting cGAS-STING pathway in gynecological cancers (**Figure 5**) (**Table 1**).

**Table 1:** Treatments against cGAS-STING pathway in gynecological cancers.

Treatment name	Drug type	Cancer Type and Cell Lines	Model (*in vivo*/*in vitro*)	Effects on cGAS-STING Pathway	Highlights	Ref.
Immunotherapy						
STING Agonist + PD-1 Checkpoint Blockade + Carboplatin	Immunotherapy + Chemotherapy	High-Grade Serous Ovarian Cancer (HGSC), ID8-Trp53−/− cells	*In vivo* (mouse model)	Activates cGAS-STING pathway, increasing type I interferon (IFN) response and antigen presentation	Combination therapy led to enhanced tumor infiltration of CD8+ T cells, significantly decreased tumor burden and ascites, and the longest survival in treated mice. Synergistic effects observed as STING agonist converted “cold” tumors (low immune activity) into “hot” tumors (high immune cell infiltration), enhancing carboplatin efficacy and immune checkpoint blockade response.	(85)
Liposomal Low-Dose Cytarabine (Ara-C/DS)	Chemotherapeutic agent in liposomal delivery	Ovarian cancer (A2780, A2780R) and Colorectal cancer (HT-29, HCT 116)	*In vitro* (cell lines) and ex vivo (PBMCs from cancer patients)	Activates cGAS-STING pathway through DNA double-strand breaks, leading to phosphorylation of STING, TBK1, and IRF3	Enhances tumor immunogenicity by increasing immune ligands (MHC I, MICA/B, ULBP2/5/6) on cancer cells, promotes cytotoxic T cell and NK cell activation; supports immune clearance of tumors.	(86)
Mn2+ and IL-12 Combination Therapy	Metal ion (Mn2+) and Cytokine (IL-12)	Ovarian cancer (ID8 cell line)	*In vitro* (cell culture), *In vivo* (murine model)	Mn2+ activates STING pathway, enhancing type I IFN production and proinflammatory M1 macrophage polarization	The combination of Mn2+ and IL-12 effectively reprograms the ovarian tumor microenvironment (TME) from an immunosuppressive “cold” state to an immune-active “hot” state. Mn2+ activates the STING pathway, promoting type I IFN production and enhancing the antigen-presenting ability of macrophages, which shifts them toward a proinflammatory M1 phenotype. IL-12 complements this by enhancing T-cell activation, improving antigen presentation, and increasing infiltration of cytotoxic CD8+ and helper CD4+ T cells into the tumor. This coordinated immune activation leads to reduced tumor growth, increased T-cell-mediated cytotoxicity, and the formation of memory T cells, supporting long-term tumor resistance. The combined therapy also demonstrates a more favorable safety profile by mitigating IL-12-associated IFN-γ toxicity, highlighting its potential for safe, durable cancer immunotherapy.	(87)
MSA-2 (STING Agonist) + Anti-PD-1	STING agonist and PD-1 inhibitor	Cervical cancer (U14, TC-1)	*In vitro* (cell analysis), *In vivo* (murine model)	Activates STING pathway, increases immune cell infiltration, and enhances CD8+ T cell and NK cell responses	MSA-2, an oral STING agonist, transforms the cervical cancer immune microenvironment from an immune-suppressive state to an immune-active one, enhancing tumor visibility to immune cells. MSA-2 upregulates STING downstream genes like CCL5, CXCL9, and CXCL10, which correlate with better prognosis and higher immune infiltration, specifically recruiting CD8+ T cells, NK cells, M1-type macrophages, and T follicular helper cells. The addition of anti-PD-1 therapy to MSA-2 further potentiates immune responses, overcoming immune checkpoint inhibitor resistance by enhancing T-cell-driven tumor clearance. The treatment strategy significantly suppresses tumor growth, prolongs survival, and induces tumor cell apoptosis without evident immune-related toxicities, positioning MSA-2 as a promising candidate for clinical trials in cervical cancer.	(88)
Nanoparticles Therapy						
Gas-Amplified Metalloimmunotherapy	PEGylated Mn-doped calcium sulfide nanoparticles (MCSP)	Cervical Cancer (U14 cells)	Both *in vivo* and *in vitro*	Activates cGAS-STING pathway through Mn²^+^ release and H_2_S-induced mitochondrial DNA release	Dual activation of pyroptosis and cGAS-STING pathway enhances both innate and adaptive immunity; H_2_S disrupts calcium homeostasis, leading to pyroptosis and immune activation. Synergistic with PD-1 blockade for tumor inhibition.	(92)
Targeted Therapy						
PARPi (Niraparib)	PARP Inhibitor	HRD-positive ovarian cancer (e.g., OVCAR5, SKOV3)	*In vivo* & *in vitro*	Induces limited activation of cGAS-STING on its own	PARPi alone has limited efficacy in promoting immunogenicity for HRD ovarian cancer; however, it sensitizes tumor cells to DNA damage and may trigger low-level immune responses.	(94)
HDACi (Entinostat)	HDAC Inhibitor	HRD-positive ovarian cancer (e.g., OVCAR5, SKOV3)	*In vivo* & *in vitro*	Promotes cGAS-STING pathway activation	HDAC inhibition elevates histone acetylation around HRD-EXCUTE hub genes, promoting immune gene expression and enhancing tumor immunogenicity, especially when combined with PARPi.	
PARPi + HDACi (Niraparib + Entinostat)	PARP Inhibitor + HDAC Inhibitor	HRD-positive ovarian cancer (e.g., OVCAR5, SKOV3)	*In vivo* & *in vitro*	Strong activation of cGAS-STING pathway, increased histone acetylation	Synergistically promotes HRD-EXCUTE expression, intensifies tumor mutation burden, increases immune cell infiltration, and remodels the immune-inflamed microenvironment.	
PARPi + ICB (Niraparib + anti-PD-1)	PARP Inhibitor + Immune Checkpoint Blockade (ICB)	HRD-positive ovarian cancer (e.g., Trp53-/-Brca2-/- ID8-Luc cells)	*In vivo*	Moderate activation of cGAS-STING	Enhances anti-tumor immunity, but overall response rates remain lower than expected. Only some HRD ovarian cancer patients benefit significantly, suggesting need for additional immune pathway activation.	
Triple Combination (PARPi + HDACi + ICB)	PARP Inhibitor + HDAC Inhibitor + Immune Checkpoint Blockade (anti-PD-1)	HRD-positive ovarian cancer (e.g., Trp53-/-Brca2-/- ID8-Luc cells)	*In vivo*	Substantial activation of cGAS-STING, enhanced immune cell infiltration	Demonstrates superior anti-tumor efficacy by inflaming the tumor microenvironment, enhancing immune cell presence, and promoting tumor immunogenicity, effectively overcoming immunotherapeutic resistance in HRD-EXCUTE-negative HRD ovarian cancer models.	
Niraparib	PARP Inhibitor	Ovarian cancer (SKOV3, UWB1.289)	*In vivo* (mice) & *in vitro*	Activates cGAS-STING pathway, upregulating IFN-β, CCL5, and CXCL10	Alone, Niraparib increases PD-L1 expression in ovarian cancer cells and enhances CD8+ T cell recruitment, activating an immune response via cGAS-STING, but is limited by immunosuppressive feedback from PD-L1.	(95)
PD-L1 Blockade (anti-PD-L1)	Immune Checkpoint Blockade	Ovarian cancer (ID8 cells in C57BL/6 mice)	*In vivo*	Not applicable	PD-L1 blockade alone moderately inhibits tumor growth by preventing PD-L1 mediated T cell suppression but is limited without increased immunogenicity or immune cell infiltration.	
Niraparib + PD-L1 Blockade	PARP Inhibitor + Immune Checkpoint Blockade	Ovarian cancer (ID8, SKOV3, UWB1.289)	*In vivo* (mice) & *in vitro*	Strong activation of cGAS-STING pathway, with increased IFN-β, CCL5, and CXCL10	The combination shows a significant anti-tumor effect by increasing CD8+ T cell infiltration, enhancing IFN-γ production, and sustaining immune activation in the tumor microenvironment, resulting in suppressed tumor growth.	
CDC7 Inhibition + PARP Inhibitor (Olaparib)	CDC7 inhibitor (XL413), PARP inhibitor (Olaparib)	Advanced Ovarian Cancer (e.g., OVCAR5, OVCAR8, ID8, HGS1, PDX-POVC8)	Both *in vivo* (mouse models) and *in vitro* (cell cultures)	Induces DNA damage and replication stress, leading to cytoplasmic DNA accumulation, which activates the cGAS-STING pathway. This stimulates type-I interferon signaling and boosts CD8+ T cell response.	Combination treatment overcomes PARPi resistance in ovarian cancer by enhancing DNA damage and immune response through cGAS-STING activation, increasing CD8+ T cell infiltration, and improving tumor suppression. Shows promise for broader therapeutic impact.	(96)
CX-5461	RNA Polymerase I Inhibitor	Ovarian cancer (HeyA8, COV362)	*In vivo* (mouse xenograft) & *in vitro*	Activates cGAS-STING pathway, leading to cytosolic DNA accumulation, phosphorylation of IRF3, and production of type I IFNs (IFN-β, CXCL10, IL-6)	CX-5461 induces cytosolic DNA buildup and STING activation, initiating a type I interferon response. It has potential to enhance immune responses and sensitize chemoresistant ovarian cancer cells to immunotherapy when combined with checkpoint inhibitors.	(97)
WEE1 Inhibitor (WEE1i) + ATR Inhibitor (ATRi) + PD-L1 Blockade	WEE1 kinase inhibitor (AZD1775), ATR inhibitor (AZD6738), PD-L1 immune checkpoint inhibitor	Ovarian Cancer (e.g., ID8, OVCAR8, CT26)	Both *in vivo* (mouse models) and *in vitro* (cell cultures)	Induces accumulation of cytoplasmic dsDNA, activating the cGAS-STING pathway, which triggers type I interferon production and enhances CD8+ T cell response	Combination therapy increases DNA damage and immune activation, while PD-L1 blockade prevents immune suppression, significantly improving tumor suppression in ovarian cancer	(98)
CDK12-IN-3 + Olaparib	CDK12 Inhibitor + PARP Inhibitor	Ovarian Cancer (SKOV3, OVCAR3)	*In vitro* (cell lines, PDOs), *In vivo* (xenograft)	Promotes nuclear relocalization of cGAS, inhibits HR repair via cGAS nuclear activity, genomic instability increases	Combination disrupts PARP1-Ku80 complex, enhances NHEJ over HR in G2, increases DNA double-strand breaks, cell cycle arrest, and induces persistent apoptosis, offering an effective approach for HR-proficient ovarian cancers.	(99)
Millepachine (MIL)	Topoisomerase II Inhibitor	Ovarian Cancer (SK-OV-3, A2780S)	*In vitro* (cell lines), *In vivo* (xenograft)	Affects IKK	MIL induces DNA damage through topoisomerase II inhibition, activating the NF-κB pathway in a pro-apoptotic role; it enhances ATM/IKK/NF-κB signaling, leading to apoptosis even in drug-resistant ovarian cancer models.	(100)
NH14	Small-molecule urea derivative	Ovarian cancer, SKOV3	*In vitro*	Inhibition of NF-κB signaling reduces inflammation; no direct activation of cGAS-STING	NH14 inhibits IKKα/β, which leads to reduced NF-κB activity, thereby suppressing inflammation. Potential anti-cancer effects in ovarian cancer cells.	(101)
Virotherapy						
Oncolytic HSV (RAMBO) + Cisplatin	Oncolytic Virus + Chemotherapy	Platinum-resistant Ovarian Cancer (TR127, TR182, OVTOKO, ID8)	*In vitro* and *in vivo*	Activates cGAS-STING pathway via DNA damage from retained cisplatin, leading to IFN production and enhanced immune activation	oHSV disrupts vesicular trafficking, increasing intracellular cisplatin retention and DNA damage, activating the cGAS-STING pathway and promoting immune response; enhanced antitumor immunity when combined with anti-PD-1 checkpoint inhibition.	(104)
E7GRG + 2′3′-cGAMP + CpG-C	Therapeutic vaccine (Mutated E7 protein + STING and TLR9 agonists)	HPV-related tumors (TC-1 cell line)	*In vivo* (mouse model)	Activates STING pathway, enhancing IFN-I and immune cell activation	Co-administration of 2′3′-cGAMP and CpG-C with the mutated E7 protein (E7GRG) induced strong Th1-type immune response, promoting CD8+ T cell and NK cell responses, leading to significant tumor growth inhibition in HPV-infected mice.	(105)
Bacterial Therapy						
*Lactobacillus gasseri* LGV03	Probiotic strain	HPV-positive cervical cancer (Ect1/E6E7, CaSki cells)	*In vivo* (zebrafish) and *in vitro*	Enhances type I IFN production by modulating IRF3 pathway and attenuates NF-κB-mediated inflammation to prevent excessive immune response	Selective immune modulation targeting HPV-positive cells; suppresses cervical cancer cell proliferation; no inflammatory response observed *in vivo*.	(106)
Mesenchymal Stem Cell Therapy						
Multi-transgene MSC Therapy	MSCs modified to deliver CDUPRT (prodrug-converting enzyme) and IFNb	Ovarian and Colorectal Cancer; ES2, HT-29, Colo-205	*In vitro* and *in vivo* (mouse model)	Activates cGAS-STING pathway, enhancing immune recognition and response	Co-expression of CDUPRT and IFNb increases anti-tumor activity through local 5FU production and immune modulation, transforming immune-suppressive tumors to immune-responsive.	(109)
Natural Products						
Cucurbitacin B (CuB)	Natural compound	Cisplatin-resistant ovarian cancer (A2780-DDP, C13*)	Both *in vivo* (xenograft mice) and *in vitro* (cell culture)	Activates cGAS, leading to immune response and DNA damage-induced apoptosis	Inhibits PI3K/Akt/mTOR pathway; enhances cisplatin sensitivity; promotes apoptosis and anti-tumor immunity	(114)
Wentilactone A (WA)	Natural compound from marine algae	Cisplatin-resistant ovarian cancer (SKOV3, SKOV3ip1, OVCA429, OVCA433, Hey, Hey-A8 (A8), A2780, A2780cis)	Both *in vivo* (xenograft mouse model) and *in vitro* (cell culture)	Inhibits NF-κB phosphorylation by blocking IKK/IκB, reducing ECM1 expression	Reverses cisplatin resistance in ovarian cancer by inhibiting NF-κB/ECM1 signaling; suppresses the transformation of normal fibroblasts (NFs) into cancer-associated fibroblasts (CAFs), thereby targeting both tumor cells and tumor microenvironment	(115)
Tectorigenin + Paclitaxel	Natural compound + Chemotherapy	Ovarian cancer (MPSC1TR, A2780TR, SKOV3TR)	*in vitro*	Downregulates Akt/NF-κB pathway (indirect)	Sensitizes resistant ovarian cancer cells, inducing apoptosis by inhibiting NF-κB and Akt signaling pathways​	(117)
Clinical Trials						
Rucaparib + Atezolizumab	PARP Inhibitor (Rucaparib) + Immune Checkpoint Inhibitor (Atezolizumab)	Advanced BRCA-mutated ovarian cancer	*In vivo* (patients)	Rucaparib treatment led to increased DNA damage and cytosolic DNA, activating the cGAS-STING pathway. This pathway activation boosted type I interferons and CD8+ T-cell activity, enhancing the immune response. Combining with atezolizumab further amplified this effect.	The combination therapy demonstrated acceptable safety and a 60% objective response rate (ORR) in BRCA-mutated ovarian cancer patients, including partial and complete responses. Higher baseline PD-L1 expression and CD8+ T-cell infiltration were associated with responders, highlighting potential biomarkers for treatment selection.	(118)
Prexasertib	CHK1 Inhibitor (CHK1i)	Recurrent BRCA wild-type high-grade serous ovarian cancer (HGSOC)	*In vitro* (blood/tissue samples) and *in vivo* (patients)	Prexasertib treatment increased expression of TBK1, a downstream activator in the cGAS-STING pathway, suggesting enhanced innate immune activation. This pathway potentially contributes to tumor immunogenicity by stimulating type I interferons and promoting immune cell recruitment to tumor sites.	Prexasertib treatment induced DNA damage in immune cells, increased immunocompetent monocytes, and activated the cGAS-STING pathway (via TBK1). These effects were linked with improved progression-free survival, highlighting the potential for CHK1 inhibition to enhance innate and adaptive immune responses in HGSOC.	(119)

### Immunotherapy

The cGAS-STING pathway is vital in cancer immunotherapy, acting as a sensor for cytosolic DNA and triggering immune responses to detect and combat tumor cells. When activated, it promotes interferon production and other immune-stimulatory molecules, transforming the tumor microenvironment to enhance immune cell infiltration and T cell activity. Researchers are developing STING agonists to amplify these immune responses, particularly in combination with therapies like immune checkpoint inhibitors and cancer vaccines (17).

#### cGAS-STING in immunotherapy of ovarian cancer

In HGSC, combining PD-1 immune checkpoint blockade with STING agonist therapy enhances carboplatin chemotherapy efficacy. This combination targets the immunosuppressive tumor microenvironment, especially in patients resistant to conventional chemotherapy. The STING agonist activates the innate immune system, increasing type I interferon production and antigen presentation, which recruits and activates CD8+ T cells. This immune activation primes the tumor for a better response to PD-1 blockade, preventing immune evasion by inhibiting the PD-1 pathway. The combination boosts anti-tumor immunity, reducing tumor burden and extending survival in preclinical models. It converts “cold” tumors into “hot” tumors, making the cancer more vulnerable to carboplatin and improving patient outcomes (85). Liposomal low-dose cytarabine (Ara-C) offers a promising treatment for ovarian cancer by activating the cGAS-STING pathway, a key component of cancer immunotherapy. Encapsulated in a mannosylated cationic liposomal delivery system, Ara-C enhances cellular delivery and stability, inducing DNA-DSB in ovarian cancer cells with minimal toxicity. This triggers the cGAS-STING pathway, increasing type I interferon production and upregulating immune ligands such as MHC I and NKG2D ligands. These changes improve the tumor’s immunogenicity, converting the immunosuppressive tumor microenvironment (TME) into a more inflamed, “hot” state, which recruits and activates cytotoxic T lymphocytes (CTLs) and natural killer (NK) cells. Ara-C/DS thus promotes targeted immune attacks on ovarian cancer cells, showing promise for immune-resistant tumors (86). The combination of Mn2+ and IL-12 shifts the ovarian TME from immunosuppressive to immune-active by activating innate and adaptive immunity. Mn2+ enhances the STING pathway, driving type I interferon production and polarizing macrophages to the proinflammatory M1 type for antitumor immunity. IL-12 boosts antigen presentation, T-cell activation, and infiltration of CD4+ and CD8+ T cells into tumors. Together, they enhance macrophage-mediated tumor cell phagocytosis and sustain memory T cell responses. This strategy converts “cold” tumors into “hot” tumors, increasing immune activity, reducing tumor growth, and improving long-term resistance to ovarian cancer (87).

#### cGAS-STING in immunotherapy of cervical cancer

The STING agonist MSA-2 activates the cervical cancer microenvironment by enhancing CD8+ T cells, NK cells, and M1 macrophages critical for antitumor immunity. It upregulates STING-related genes (CCL5, CXCL9, CXCL10), boosting immune cell infiltration and intercellular communication. By shifting the TME to an immune-responsive state, MSA-2 primes immune cells for a stronger response. Combined with anti-PD-1 therapy, it overcomes resistance to immune checkpoint inhibitors, improving tumor suppression and survival in preclinical models. This synergy highlights MSA-2’s potential as an adjuvant for resistant cervical cancer cases (88). Nanoparticles are tiny materials, often ranging from 1 to 100 nanometers in size, engineered for various applications, including cancer therapy, due to their unique physical and chemical properties (89–91). Gas-Amplified Metalloimmunotherapy enhances immune responses against cervical cancer by inducing pyroptosis and activating the STING pathway to remodel the tumor’s immunosuppressive microenvironment. PEGylated manganese-doped calcium sulfide nanoparticles (MCSP) release Ca²^+^, Mn²^+^, and H_2_S at the tumor site. H_2_S disrupts the cancer cell’s calcium-buffering system, causing calcium overload that triggers pyroptosis, releasing tumor antigens and activating immunity. Additionally, H_2_S-induced mitochondrial dysfunction releases DNA, which, with Mn²^+^, activates the cGAS-STING pathway, boosting dendritic cell activation and antigen presentation. This dual mechanism enhances immune responses and improves immunotherapy, especially with PD-1 inhibitors (92).

#### Targeted therapy in ovarian cancer

Targeted therapy in ovarian cancer aims to improve outcomes by addressing molecular complexities and overcoming chemotherapy resistance, especially in high-grade serous ovarian cancer (HGSOC). PARP inhibitors (e.g., olaparib, niraparib, rucaparib) target DNA repair pathways, significantly benefiting patients with BRCA mutations. Angiogenesis inhibitors like bevacizumab enhance chemotherapy by restricting tumor blood supply. Emerging therapies target lipid metabolism, receptor tyrosine kinases, and immune checkpoints, though immune checkpoint inhibitors alone show limited success. Biomarker-driven treatments, such as folate receptor alpha (Frα) targeting, show promise in platinum-resistant cases. Despite progress, resistance remains a challenge, highlighting the need for ongoing research to refine therapies and improve survival rates (93). Combining PARP inhibitors like Niraparib and Olaparib with HDAC inhibitors such as Entinostat enhances the immunogenicity of HRD ovarian cancer by upregulating the HRD-EXCUTE phenotype. This biomarker, defined by 15 immune-activation genes, fosters an “immune-inflamed” tumor microenvironment, attracting CD8+ T cells and macrophages. The combination therapy activates the cGAS-STING pathway and increases histone acetylation around HRD-EXCUTE genes, boosting immune signaling. In preclinical models, it significantly delayed tumor growth and improved survival compared to individual treatments. Combined with immune checkpoint inhibitors, this approach also overcame immunotherapy resistance, highlighting its potential in HRD-EXCUTE-positive ovarian cancer (94). Similarly, Niraparib, a PARP inhibitor, enhances anti-tumor effects when combined with PD-L1 blockade by modulating immune responses in ovarian cancer. It increases PD-L1 expression on tumor cells but activates the cGAS/STING pathway, triggering cytokine production (e.g., CCL5, CXCL10) that recruits and activates CD8+ T cells. These T cells target and kill cancer cells. In mouse models, this combination significantly inhibits tumor growth more effectively than either treatment alone by boosting T cell infiltration and activation, presenting a promising strategy for ovarian cancer therapy (95). CDC7, a serine-threonine kinase critical for DNA replication and fork stability, plays a key role in advanced ovarian cancer. Inhibiting CDC7 (CDC7i) enhances the efficacy of PARP inhibitors (PARPi) like Olaparib, particularly in BRCA-mutated or homologous recombination-deficient cancers. CDC7 inhibition disrupts DNA replication, increasing DNA damage and replication stress, which activates the cGAS/STING pathway and induces a type-I interferon response, boosting antitumor immunity. Combined, CDC7i and PARPi amplify DNA damage, trigger cell cycle arrest, and enhance immune activation, presenting a potent strategy for PARPi-resistant or advanced ovarian cancer (96).

CX-5461, an RNA polymerase I inhibitor, shows promise in chemoresistant cancers, including ovarian cancer. By inhibiting ribosomal RNA synthesis, it triggers DNA damage via ATM and ATR kinases, independent of p53. In ovarian cancer cells, CX-5461 induces cytosolic DNA accumulation, activating the cGAS-STING pathway. This activation leads to IRF3 phosphorylation and production of type I interferons (e.g., IFN-β) and inflammatory cytokines (e.g., CXCL10, IL-6), enhancing the tumor’s immune profile. CX-5461 holds potential in combination therapies with immune checkpoint inhibitors, improving immunotherapy outcomes in resistant ovarian cancers (97).

WEE1i, a WEE1 kinase inhibitor, disrupts the cell cycle at the G2/M checkpoint, forcing cancer cells with DNA damage into premature mitosis, leading to cell death. In ovarian cancer, WEE1 inhibition combined with ATR inhibitors (ATRi) increases DNA damage and stress, triggering cytoplasmic dsDNA accumulation and activating the cGAS-STING pathway. This activation stimulates type I interferons and recruits CD8+ T cells, boosting anti-tumor immunity. However, the resulting interferon response upregulates PD-L1 on tumor cells, which suppresses T cell activity. Combining PD-L1 blockade with WEE1i and ATRi enhances the immune response, improving tumor suppression and immune-mediated cancer cell targeting (98). CDK12-IN-3 is a selective inhibitor of cyclin-dependent kinase 12 (CDK12), which regulates DNA repair, particularly homologous recombination (HR). When combined with Olaparib, a PARP inhibitor, CDK12-IN-3 further disrupts DNA repair by inhibiting HR, promoting genomic instability in ovarian cancer cells. This combination increases non-homologous end joining (NHEJ), an error-prone repair pathway, while reducing HR activity. CDK12-IN-3 impairs Ku80 phosphorylation and PARylation, disrupting the PARP1-Ku80 complex and relocating cGAS to the nucleus, amplifying genomic instability. The resulting DNA damage induces cell cycle arrest and cell death, offering a promising strategy for HR-proficient ovarian cancers resistant to PARP inhibitors (99).

Millepachine (MIL), a chalcone from Millettia pachycarpa, exhibits potent anti-cancer effects in ovarian cancer. As a topoisomerase II inhibitor, MIL stabilizes the DNA-topoisomerase II complex, causing DSBs that cancer cells struggle to repair. This damage activates the ATM pathway, which phosphorylates IKKα/β, further activating NF-κB (p65) and promoting its nuclear translocation. In this context, NF-κB acts pro-apoptotically, upregulating apoptotic markers like Bax and cytochrome C release, while downregulating anti-apoptotic proteins like Bcl-2, leading to cell death. MIL shows efficacy in both standard and drug-resistant ovarian cancer models, making it a promising therapeutic agent (100). Similarly, NH14 (N-(4-ethylphenyl)-N′-phenylurea) is a small-molecule inhibitor targeting the inflammatory response mediated by IKKα/β in the NF-κB pathway. It inhibits NF-κB activation by blocking IKKα/β downstream of inflammatory receptors like TLR2, TLR4, TNF-R, and IL-1R. In ovarian cancer cells, NH14 reduces NF-κB activity, preventing IκBα degradation and decreasing TNFα production after inflammation. This inhibition limits cell migration and wound healing, key processes for cancer proliferation and metastasis. Docking studies show NH14 binds to the hinge region of IKKβ, positioning it as a promising candidate for selective anti-inflammatory and anti-cancer therapies targeting IKK pathways (101).

#### Virotherapy in ovarian cancer

Oncolytic virotherapy is an emerging ovarian cancer treatment using viruses that selectively infect and destroy cancer cells while sparing normal ones. These oncolytic viruses (OVs) exploit cancer cells’ genetic and immune vulnerabilities, replicating within and killing tumors while often triggering an immune response. Viruses like adenovirus, reovirus, and measles virus have shown promising results in preclinical and early clinical studies. However, challenges include improving targeted delivery to tumors, overcoming resistance in cancer-initiating cells, and managing immune responses. Combining OVs with chemotherapy has shown synergistic effects, and next-generation OVs are being developed for enhanced targeting and immune stimulation. Oncolytic virotherapy holds significant potential for treating recurrent or drug-resistant ovarian cancer [Reviewed by (102)]. Ovarian cancer cells often have defective STING signaling, which is important for immune responses. The STING pathway detects cytosolic DNA, including viral and damaged self-DNA, triggering immune reactions. However, many ovarian cancer cells exhibit impaired STING signaling, often due to epigenetic silencing, such as DNA hypermethylation of STING or its activator cGAS. This deficiency allows cancer cells to evade immune detection by suppressing cytokine production. Paradoxically, this defect makes ovarian cancer cells more vulnerable to oncolytic viruses like HSV1Δγ34.5, which can replicate more effectively in STING-deficient cells, leading to cell lysis and tumor reduction. Therefore, defective STING signaling creates a therapeutic vulnerability, making these tumors more responsive to oncolytic virotherapy (103). Oncolytic herpes simplex virus (oHSV) therapy enhances ovarian cancer cells’ sensitivity to cisplatin while triggering a strong antitumor immune response. Infection with oHSV disrupts vesicular trafficking and lysosomal pathways, which normally expel drugs like cisplatin, leading to increased cisplatin retention and DNA damage. This damage results in micronuclei formation, activating the cGAS–STING pathway and initiating inflammatory signaling that recruits NK cells and T cells. Additionally, oHSV increases PD-L1 expression on tumor cells, making them more responsive to anti-PD-1 checkpoint blockade. This combination improves chemotherapy efficacy and creates an immunogenic environment, enhancing cancer immunotherapy and overcoming chemoresistance (104).

#### Virotherapy in cervical cancer

E7GRG, a modified form of the HPV 16 E7 oncoprotein, retains immunogenicity while reducing transformation potential, making it a safer candidate for therapeutic vaccination against HPV-related cancers, such as cervical cancer. When combined with the immune-stimulating adjuvants 2′3′-cGAMP, a STING pathway activator, and CpG-C, a TLR9 agonist, it triggers a strong immune response in the tumor microenvironment. 2′3′-cGAMP activates the STING pathway, boosting IFN-I production, enhancing innate immunity, and recruiting immune cells to the tumor. CpG-C stimulates TLR9, inducing pro-inflammatory cytokines and promoting a Th1 immune response, more effective against cancer cells. This combination enhances CD8+ T cell and NK cell activity, increasing granzyme B levels and promoting tumor cell apoptosis. These mechanisms result in significant tumor growth inhibition in cervical cancer mouse models, making E7GRG with 2′3′-cGAMP and CpG-C a promising therapeutic vaccine for HPV-driven tumors (105).

#### Bacterial therapy in cervical cancer

Lactobacillus gasseri LGV03, isolated from the cervico-vaginal samples of women who cleared HPV infection, modulates epithelial innate immune responses and inhibits the growth of HPV-positive cervical cancer cells. The probiotic enhances antiviral defenses by upregulating the production of type I interferons (IFN-α and IFN-β) through the IRF3 signaling pathway, which keeps the immune system vigilant against pathogens. Simultaneously, L. gasseri LGV03 attenuates inflammatory responses by downregulating proinflammatory cytokines (IL-6, IL-8, and IL-1β) via the NF-κB pathway, helping prevent excessive inflammation. *In vivo* studies using zebrafish models demonstrated that L. gasseri LGV03 significantly suppressed the proliferation of HPV-positive cervical cancer cells, likely through an enhanced immune response, without inducing inflammation (106).

#### Mesenchymal stem cells therapy in ovarian cancer

Mesenchymal stem cells (MSCs) modified with multiple transgenes are MSCs engineered to carry and express specific therapeutic genes, enhancing their capacity to target and treat diseases like peritoneal carcinomatosis (PC) more effectively (107, 108). For ovarian cancer, MSCs can be engineered to co-express transgenes that activate chemotherapy agents and stimulate immune responses in the tumor microenvironment. MSCs expressing cytosine deaminase uracil phosphoribosyl transferase (CDUPRT) can convert the prodrug 5-flucytosine (5FC) into 5-fluorouracil (5FU) at the tumor site, reducing systemic toxicity. Additionally, co-expression of interferon-beta (IFNb) enhances immune activation via the cGAS-STING pathway, which recognizes DNA damage and triggers an immune response. This pathway can shift the tumor microenvironment from immune-suppressive to immune-responsive, aiding the immune system in targeting cancer cells. Thus, multi-transgene modified MSCs provide localized chemotherapy while stimulating a durable anti-tumor immune response, offering a promising approach for ovarian cancer treatment (109).

#### Natural products therapy in ovarian cancer

Natural and semi-synthetic compounds show promise in treating ovarian cancer by targeting multiple cancer-related processes with minimal side effects. These compounds can induce apoptosis, reduce cell proliferation, and modulate oxidative stress, controlling cancer growth. Substances like curcumin, resveratrol, and quercetin also possess anti-inflammatory and anti-metastatic properties, inhibiting pathways essential for cancer spread and blood vessel formation. Additionally, they can enhance traditional chemotherapy by reducing resistance and adverse effects, serving as complementary therapies. Semi-synthetic derivatives are being developed to improve stability, bioavailability, and efficacy. However, more clinical studies are needed to determine optimal dosing and delivery for their role in ovarian cancer treatment (110, 111). Cucurbitacin B (CuB) is a natural compound derived from cucurbitaceous plants, known for its anti-inflammatory, anti-tumor, and immune-regulatory properties (112). Recent studies indicate that CuB has the potential to overcome cisplatin resistance in ovarian cancer, a major hurdle in effective cancer treatment. CuB achieves this by inhibiting the PI3K/Akt/mTOR pathway, which is crucial for tumor cell growth, proliferation, and survival (113, 114). Additionally, CuB activates the cGAS immune pathway, inducing DNA damage in cancer cells and promoting an anti-tumor immune response. By targeting mTOR directly and enhancing cancer cell apoptosis, CuB not only inhibits tumor growth but also boosts the effectiveness of cisplatin therapy, suggesting it could be used alone or in combination with other chemotherapeutics to improve treatment outcomes in cisplatin-resistant ovarian cancer (114). Similarly, Wentilactone A (WA), a tetranorditerpenoid from marine algae and the fungal endophyte Aspergillus wentii, has shown anti-cancer properties, especially against cisplatin-resistant ovarian cancer. WA targets the NF-κB/ECM1 signaling pathway, involved in cancer cell survival and drug resistance. By inhibiting IKK/IκB phosphorylation, WA prevents NF-κB activation, reducing ECM1 expression, a key factor in cisplatin resistance and tumor progression. WA also blocks the transformation of normal fibroblasts into CAFs, which support drug resistance and tumor growth. Through these actions, WA sensitizes ovarian cancer cells to cisplatin and limits tumor-supportive changes in the microenvironment, making it a valuable adjunct in ovarian cancer therapy (115). Flavonoids are natural plant compounds with antioxidant properties, found in fruits, vegetables, and beverages like tea, known for their role in reducing inflammation and supporting overall health (116). Tectorigenin, an isoflavonoid from Pueraria thunbergiana, sensitizes paclitaxel-resistant ovarian cancer cells by promoting apoptosis. It enhances paclitaxel efficacy by downregulating the IKK pathway, part of the Akt/NF-κB signaling axis linked to drug resistance. Inhibiting IKK prevents NF-κB translocation to the nucleus, reducing survival gene expression (e.g., Bcl-2 and XIAP). This disruption of survival signals facilitates cell death in response to paclitaxel. These findings suggest that tectorigenin, by targeting chemoresistance pathways, could serve as an adjuvant to restore paclitaxel efficacy in ovarian cancer therapies (117).

## Clinical trials in targeting cGAS-STING in ovarian cancer

The COUPLET study (118) is a Phase Ib clinical trial designed to evaluate the combination of rucaparib, a PARP inhibitor (PARPi), and atezolizumab, an immune checkpoint inhibitor (ICI) targeting PD-L1, in patients with advanced ovarian cancer. The results highlighted that the combination therapy was generally well-tolerated, with common side effects including gastrointestinal issues, fatigue, and elevated liver enzymes. Importantly, patients with BRCA-mutated tumors showed notable responses, with a 60% objective response rate in ovarian cancer patients. The COUPLET study provided valuable insights into the activation of the cGAS-STING pathway in ovarian cancer when treated with a combination of rucaparib and atezolizumab. In responders, cGAS-STING pathway activation increased notably during the initial rucaparib run-in phase, which preceded the addition of atezolizumab. This pathway, responsible for sensing cytosolic DNA fragments resulting from DNA damage, plays a critical role in stimulating anti-tumor immune responses. Specifically, DNA damage induced by PARP inhibition with rucaparib led to an accumulation of cytosolic DNA, which activated cGAS. Once activated, cGAS catalyzed the formation of cGAMP, which in turn stimulated the STING protein pathway. This activation of STING promoted the release of type I interferons and other inflammatory signals, which enhanced immune cell recruitment, particularly of CD8+ T-cells, to the tumor microenvironment. Consequently, increased T-cell activity and a more robust immune response were observed in patients who responded to treatment. This mechanism was particularly evident in patients with BRCA-mutated ovarian cancer, where deficient DNA repair capacity (due to BRCA mutation) led to enhanced effects of PARP inhibition and subsequently amplified the immune-stimulating action of the cGAS-STING pathway. In contrast, non-responders showed less activation of this pathway, suggesting that cGAS-STING activation may correlate with treatment efficacy in PARP-inhibitor and immune-checkpoint inhibitor combinations. This finding underscores the potential for cGAS-STING activation as a biomarker for response and provides a basis for further exploration of PARP-ICI combinations to boost anti-tumor immunity in ovarian cancer (118). Similarly, a phase II study investigating the effects of prexasertib, a CHK1 inhibitor (CHK1i), in patients with recurrent BRCA wild-type (BRCAwt)-HGSOC demonstrated promising immune-modulatory and anti-tumor effects. The study observed significant DNA damage in immune cells, as shown by increased γ-H2AX staining, and a reduction in peripheral CD4+ and CD8+ T-cell populations, indicating lymphodepletion. Notably, treatment with CHK1i led to an increase in monocytes, particularly those expressing the immunocompetence marker HLA-DR, which correlated with improved progression-free survival (PFS). Additionally, tumor biopsies revealed an increase in naïve B-cells and resting memory T-cells, suggesting adaptive immune engagement, while higher infiltration of regulatory T-cells (T-regs) was associated with worse PFS, indicating a potential compensatory immunosuppressive response. Increased expression of TBK1, a protein in the STING pathway linked to innate immunity, was also associated with longer PFS, supporting a role for CHK1i in enhancing immune activation. These findings suggest that CHK1 inhibition with prexasertib may enhance both innate and adaptive immune responses in HGSOC, highlighting potential benefits of combining CHK1i with immunotherapy in future treatments (119).

## Conclusion and future perspectives

In conclusion, the cGAS-STING pathway represents a significant advancement in understanding immune responses within the context of gynecological cancers, such as ovarian, cervical, and endometrial cancers. This pathway’s ability to detect cytoplasmic DNA and trigger immune responses highlights its dual role in tumor suppression and, in some cases, promotion of tumor growth due to inflammation-induced mechanisms. Through a detailed exploration of molecular mechanisms, this study underscores the pathway’s potential as a target for innovative therapies, particularly in overcoming resistance to chemotherapy and immunotherapy. Inducing cGAS-STING activation can significantly enhance outcomes from chemotherapy and immunotherapy, amplifying the body’s ability to recognize and target cancer cells effectively. This pathway is especially promising in BRCA-mutated cancers, such as ovarian cancer, where its activation exposes vulnerabilities that can be therapeutically exploited. Additionally, in HPV-induced cancers, like cervical cancer, cGAS-STING represents a critical target, given its role in immune surveillance and response against virus-associated tumor growth. Combining cGAS-STING agonists with PARP inhibitors (PARPi) presents a potent approach, particularly in ovarian cancer, where it can create a synergistic antitumor effect. This combination holds even greater potential when paired with oncolytic viruses, which can further stimulate immune responses and enhance therapeutic efficacy. Such integrative strategies could yield transformative results, especially in challenging cases of therapy-resistant ovarian cancer. STING agonists have shown promise in combating late-stage gynecological cancers, but their efficacy is influenced by the complexities of the tumor microenvironment. In cervical cancer, STING activation through agonists like ADU-S100 and MSA-2 enhances CD8+ T-cell infiltration and boosts immune activity, leading to significant tumor size reduction in preclinical models. However, in ovarian cancer, the effects of STING activation are dual-faceted: while it promotes immune cell infiltration and type I interferon responses, it simultaneously upregulates VEGF-A, fostering angiogenesis and immune resistance. These challenges underscore the immunosuppressive nature of advanced tumors. Strategies such as combining STING agonists with immune checkpoint inhibitors (e.g., anti-PD-1 therapies) or chemotherapy have demonstrated enhanced therapeutic outcomes. These combinations help reprogram the tumor microenvironment from immunosuppressive to immunogenic, improving immune cell recruitment and activity against the tumor. Thus, while STING agonists hold substantial potential, their integration into multimodal treatment regimens is critical to counteracting the adaptive resistance mechanisms of late-stage tumors. Despite these promising insights, gaps remain in our understanding and application of cGAS-STING targeting across gynecological cancers. Research has disproportionately focused on ovarian cancer, leaving cervical and endometrial cancers less explored in this context. Addressing these gaps is essential to fully harness the potential of cGAS-STING in gynecological oncology. In particular, endometrial cancer remains understudied, highlighting an urgent need for more research and clinical trials to validate cGAS-STING targeting strategies in this cancer type. Additionally, clinical trials in cervical and endometrial cancers are essential to establish robust, evidence-based therapeutic protocols. Further, while advancements in drug-based targeting of cGAS-STING are notable, less attention has been given to alternative approaches such as natural products, nanoparticles, and mesenchymal stem cell-based therapies. Exploring these options could broaden the therapeutic landscape and offer innovative, potentially less toxic methods for activating the pathway. Moreover, evaluating the role of cGAS-STING in enhancing radiotherapy outcomes could provide valuable insights, particularly for cancers where traditional treatments have limitations. Cutting-edge technologies like single-cell RNA sequencing and CRISPR-based functional genomics hold immense promise for decoding the complexities of the cGAS/STING pathway in gynecological cancers. Single-cell analysis provides unparalleled resolution in mapping tumor heterogeneity, enabling the discovery of rare cell populations, such as immune-evasive cancer cells or immunosuppressive elements within the microenvironment, that influence cGAS/STING activity. This approach reveals context-specific regulatory dynamics, including variations in pathway activation across cell types or suppression due to tumor-induced epigenetic alterations. Simultaneously, CRISPR screening enables high-throughput identification of critical genetic and epigenetic regulators of cGAS/STING signaling. These screens can uncover novel factors that either amplify anti-tumor immune responses or facilitate immune evasion. Together, these technologies offer actionable insights into pathway mechanisms, aiding the development of targeted therapies and optimizing combination treatments, such as pairing STING agonists with immune checkpoint inhibitors or DNA-damage-based approaches. Integrating these tools with spatial transcriptomics and proteomics could map the spatial and temporal dimensions of cGAS/STING activity in the tumor microenvironment, deepening our understanding of its dual roles and unlocking new therapeutic opportunities. In summary, the cGAS-STING pathway holds transformative potential in gynecological cancer therapy, but more diverse and expansive studies are required to fully leverage its therapeutic benefits across all gynecological cancers. By addressing these research gaps and expanding therapeutic approaches, the field can advance toward more effective, personalized treatments that improve patient outcomes in these challenging cancer types.
